# Distinctive Effects of D1 and D2 Receptor Agonists on Cortico-Basal Ganglia Oscillations in a Rodent Model of L-DOPA-Induced Dyskinesia

**DOI:** 10.1007/s13311-022-01309-5

**Published:** 2022-11-07

**Authors:** Katrine Skovgård, Sebastian A. Barrientos, Per Petersson, Pär Halje, M. Angela Cenci

**Affiliations:** 1 Basal Ganglia Pathophysiology Unit, Department of Experimental Medical Science, Lund University, BMC A13, 221 84 Lund, Sweden; 2 The Group for Integrative Neurophysiology and Neurotechnology, Department of Experimental Medical Science, Lund University, Lund, Sweden; 3 Department of Integrative Medical Biology, Umeå University, Umeå, Sweden

**Keywords:** Parkinson’s disease, Dyskinesia, Oscillations, Local field potential, Dopamine agonist, Basal ganglia

## Abstract

L-DOPA-induced dyskinesia (LID) in Parkinson’s disease has been linked to oscillatory neuronal activities in the cortico-basal ganglia network. We set out to examine the pattern of cortico-basal ganglia oscillations induced by selective agonists of D1 and D2 receptors in a rat model of LID. Local field potentials were recorded in freely moving rats using large-scale electrodes targeting three motor cortical regions, dorsomedial and dorsolateral striatum, external globus pallidus, and substantial nigra pars reticulata. Abnormal involuntary movements were elicited by the D1 agonist SKF82958 or the D2 agonist sumanirole, while overall motor activity was quantified using video analysis (DeepLabCut). Both SKF82958 and sumanirole induced dyskinesia, although with significant differences in temporal course, overall severity, and body distribution. The D1 agonist induced prominent narrowband oscillations in the high gamma range (70–110 Hz) in all recorded structures except for the nigra reticulata. Additionally, the D1 agonist induced strong functional connectivity between the recorded structures and the phase analysis revealed that the primary motor cortex (forelimb area) was leading a supplementary motor area and striatum. Following treatment with the D2 agonist, narrowband gamma oscillations were detected only in forelimb motor cortex and dorsolateral striatum, while prominent oscillations in the theta band occurred in the globus pallidus and nigra reticulata. Our results reveal that the dyskinetic effects of D1 and D2 receptor agonists are associated with distinct patterns of cortico-basal ganglia oscillations, suggesting a recruitment of partially distinct networks.

**Supplementary Information:**

The online version contains supplementary material available at 10.1007/s13311-022-01309-5.

## Introduction

Parkinson’s disease (PD) is a progressive neurodegenerative disorder characterised by a severe loss of dopaminergic innervation to cortico-basal ganglia motor networks. This leads to an emergence of oscillatory neuronal activities that are believed to disrupt the dynamic processing of movement-related information and thus generate motor symptoms [1]. Local field potential (LFP) recordings during parkinsonian hypokinetic states demonstrate a synchronised oscillatory activity in the beta range (12–35 Hz) in both PD patients [2–4] and animal models of PD [5–8]. The exaggerated beta-band activity is suppressed by dopaminergic drugs as they improve parkinsonian motor symptoms [1] (for an extensive review of oscillatory activities in PD, see [9, 10]). The dopamine precursor L-DOPA is the most efficacious symptomatic medication for PD, although it leads to a development of motor fluctuations and dyskinesia in the majority of patients (reviewed in [11]). These complications are associated with the emergence of new types of oscillatory neuronal activities in the basal ganglia. During the expression of L-DOPA-induced dyskinesia (LID), low-frequency oscillations in the theta range (5–10 Hz) have been detected in deep basal ganglia nuclei in both PD patients [12–14] and animal models of LID [15–17]. Moreover, recordings from the motor cortex have revealed a strong association between the expression of involuntary movements and characteristic narrowband gamma (NBG) oscillations, which have been detected in both the rat model of LID [18–20] and in PD patients [21]. Because of their strong association with dyskinesia, NBG oscillations have been proposed to serve as a feedback signal for adaptive deep brain stimulation (DBS) [22]. In general terms, NBG oscillations of the LFP are thought to reflect rhythmic synchronisations of transmembrane currents among populations of neurons in the recorded brain region (reviewed in [23]). However, little is known about the mechanisms through which L-DOPA elicits NBG oscillations specifically in dyskinetic subjects.

In both human PD and dopamine-denervated animals, L-DOPA exerts its motor effects by stimulating D1 and D2 receptors, which are highly expressed in the striatum but also present in other critical nodes of the cortico-basal ganglia network. In the rat motor cortex, the distribution of D1 and D2 receptors exhibits a rostral-caudal gradient of decreasing density, correlating with the distribution of dopaminergic terminals [24, 25]. In the striatum, the expression of D1 and D2 receptors is segregated between “direct pathway” and “indirect pathway” spiny projection neurons (dSPNs and iSPNs, respectively), which influence the basal ganglia output via different routes (reviewed in [26]). Thus far, the role of these main dopamine receptor systems in the expression of LID-related oscillations has only been investigated in the primary motor cortex (forelimb area) and only upon acute administration of D1 or D2 receptor agonists, which were both reported capable of inducing cortical high gamma activity [19]. We here present a comprehensive investigation of oscillatory responses to D1 vs. D2 receptor agonists utilising a technology that enables large-scale multi-structure recordings in awake behaving animals [27]. Applying this technology to a validated rat model of LID [28], we analyse LFP oscillations in both NBG and theta frequency bands from seven critical nodes of the cortico-basal ganglia motor network. Our results reveal prominent differences in the oscillatory patterns mediated by D1 vs. D2 receptors in dyskinetic animals, suggesting a recruitment of partially distinct networks.

## Materials and Methods

### Animals

Experiments were performed in adult female Sprague–Dawley rats, as in our previous studies on the subject [15, 16, 18]. Animals (purchased from Janvier Labs, France) weighed 250 g on arrival and were housed in standard cages under controlled temperature (22 °C) and humidity (50%) laboratory conditions on a 12:12 h light/dark cycle (lights on at 6:00 a.m.). Food and water were available ad libitum. All procedures were approved by the Malmö-Lund Ethical Committee on Animal Research.

### Study Design

**Fig. 1:**
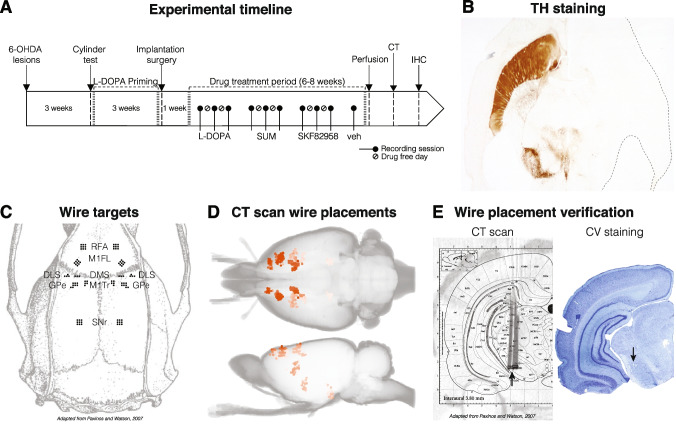
Experimental procedure and validation of wire placement. **A **Schematic representation of the study design. Rats sustained 6-OHDA lesions in the right MFB and were primed with L-DOPA (6 mg/kg s.c.) before implantation surgery. When recovered, all animals received treatment with L-DOPA, the D2R agonist sumanirole (SUM; 2 mg/kg s.c.), the D1R agonist SKF82958 (0.05 mg/kg s.c.), and vehicle (veh) with 2–3 injections per week for a total of 6–8 weeks. A drug-free break of three days was introduced between treatments. When introducing a new dyskinetic treatment, the drug was administered at least twice before recordings were recommenced to ensure a stable level of dyskinesia. Following perfusion, CT scans and immunohistochemical analysis were performed to identify the location of the tip of each wire. **B **Representative tyrosine hydroxylase (TH) immunostaining of dopaminergic neurons in a horizontal section of one animal. **C **Dorsal view of the rat skull with a constellation of wire targets. (Adapted from Paxinos and Watson [45]). **D **Horizontal (top) and sagittal (bottom) view of the location of the tip of each wire identified from CT scans. **E **Comparison between immunohistochemical analysis of CV stained brain sections and CT segmentation overlaid the corresponding atlas from Paxinos and Watson [45] for SNr of one example brain. Arrows indicate one electrode tip identified by each method. Abbreviations: computed tomography (CT), cresyl violet (CV), rostral forelimb area (RFA), primary motor cortex forelimb area (M1FL), primary motor cortex trunk area (M1Tr), dorsomedial striatum (DMS), dorsolateral striatum (DLS), globus pallidus pars externa (GPe), and substantia nigra pars reticulata (SNr)

Rats sustained unilateral 6-hydroxydopamine (6-OHDA) lesions and 3 weeks after the lesion, they were primed with L-DOPA (6 mg/kg s.c.) daily for 1 week and then every second day for 2 weeks before electrode implantation surgery (Fig. 1A). Starting 1 week from electrode implantation, all animals received treatment with L-DOPA (*n* = 9), the D2 receptor agonist sumanirole (2 mg/kg s.c., *n* = 8), and the D1 receptor agonist SKF82958 (0.05 mg/kg s.c., *n* = 9), according to the order shown in Fig. 1A, with 2–3 injections per week for a total of 6–8 weeks. Animals were recorded on vehicle treatment with saline at different time points throughout the drug treatment period (veh; vehicle; *n* = 9). Each treatment was administered every other day for 1–2 weeks and electrophysiological recordings and dyskinesia rating sessions were carried out on the days of drug challenges (Fig. 1A). However, when introducing a new treatment, a drug-free break of minimum three days was introduced between treatments and the new drug was administered at least twice before recordings were recommenced to ensure a stable level of dyskinesia. All animal were perfused and CT scans and immunohistochemical analysis were performed to identify the location of the tip of each wire (Fig. 1A–E).

### Drug Treatments

Dose–response curves were performed in a separate group of non-implanted rats prior to commencing the physiological recording study (see Supplemental Methods). The doses chosen were well tolerated (Supplemental Figs. S1 and S2) and in the range of those previously reported to produce robust behavioural effects in rodents through the indicated targets [29–34]. L-DOPA (levodopa methyl ester hydrochloride, 6 mg/kg; Sigma Aldrich AB, Sweden), sumanirole (SUM; sumanirole maleate, 2.0 mg/kg; Tocris, UK), and SKF82958 (SKF-82958 hydrobromide, 0.05 mg/kg; Tocris, UK) were given subcutaneously (s.c.). L-DOPA was co-administered with the peripheral DOPA decarboxylase inhibitor benserazide hydrochloride (12 mg/kg s.c., Sigma Aldrich AB, Sweden). All drugs used in this study were dissolved in physiological saline solution and administered in a volume of 1 ml/kg.

### 6-Hydroxydopamine Lesions and L-DOPA Priming

Unilateral nigrostriatal dopamine lesions were performed by injecting 6-OHDA into the medial forebrain bundle (MFB) according to a well-established method [35]. Briefly, rats were anaesthetised with fentanyl/medetomidine (0.21/0.21 mg/kg, intraperitoneally (i.p.); Apoteket AB, Sweden) and fixed in a stereotaxic frame (David Kopf Instruments, CA, USA). The toxin 6-OHDA hydrochloride (Sigma Aldrich AB, Sweden) was dissolved in 0.2% ascorbate-saline at a concentration of 3.5 µg/µl (free base) and injected into the MFB of the right hemisphere at the following coordinates (in mm, relative to bregma and dural surface): injection site I (2.5 µl), anterior–posterior (AP) − 4.0, medial–lateral (ML) − 1.2, dorsal–ventral (DV) − 7.8, and tooth bar in flat skull position (approximately − 4.5); and injection site II (2.0 µl), AP − 4.0, ML − 0.8, DV − 8.0, and tooth bar + 3.4 [36, 37]. A 5-µl Hamilton syringe was used for the injections that were performed at an injection rate of 1 µl/min. The needle was left in place for 2.5 min after each injection. After surgery, the anaesthesia was reversed by atipamezole hydrochloride (0.5 mg/kg, i.p.; Apoteket AB, Sweden), and buprenorphine (0.05 mg/kg, s.c.; Apoteket AB, Sweden) was administered as postoperative analgesic. The animals were given extra postoperative care for at least 5 days to facilitate their full recovery.

Three weeks after the lesion, animals with severe (> 85%) unilateral dopamine denervation were selected using the cylinder test of forelimb use asymmetry [35, 37–39]. Briefly, rats were placed individually in a glass cylinder (25 cm in diameter and 40 cm in height) and video filmed for 3–5 min. The total number of supporting wall contacts performed independently with the left and right forepaw was counted offline. Then, counts from the forepaw contralateral to the lesion were expressed as a percentage of the total number of wall contacts. Starting 3 weeks after the lesion, rats with less than 25% contralateral paw usage were primed with L-DOPA (Fig. 1A). Recording electrodes were implanted in animals that developed moderate to high levels of dyskinetic behaviours (cut-off criterion: AIM severity grade ≥ 2 on at least two AIM subtypes on most monitoring periods, as in [40]). In total, 11 animals were selected for electrode implantations surgery using these criteria.

### Abnormal Involuntary Movements (AIMs) Ratings

Dyskinesia was assessed online using both the basic AIMs scale [36] and an amplitude scale [35]. The basic scale evaluates AIM severity based on the percentage of observation time during which a dyskinetic feature is present, considering three subtypes of dyskinetic movements (axial, forelimb, and orolingual AIMs) [36]. The basic scale was combined with the AIMs amplitude scale, which evaluates the deviation of a body part from its natural resting position and the number of muscle groups visibly engaged in the dyskinetic movement [35]. Thus, all AIM subtypes were scored simultaneously with respect to their severity and amplitude following drug injection for monitoring periods of 1 min every 5 min for the first 20 min and then every 10 min for the rest of the testing session. Each of the AIM subtypes received a severity score and an amplitude score in a scale from 0 to 4 on each monitoring period. A composite AIM score was then produced by multiplying the severity score and amplitude score for each AIM subtype on each monitoring period and all AIM scores were summed to obtain a global measure of dyskinesia for each testing session (global AIM score).

### Recording Electrodes

A recording implant of 94 insulated tungsten wires (33 µm diameter; California Fine Wire Company, CA, USA) was built as previously described [27]. The microwires were arranged into 14 electrode bundles (7 per hemisphere) with 250 µm spacing between wires in each horizontal dimension and a wire length corresponding to the depth of each recording target (see Table 1 for coordinates and main references) (Fig. 1C). A 125-µm silver wire (Advent Research Materials Ltd, UK) was used for ground connection. All wires from each hemisphere were connected to a custom-designed printed circuit board with silver conductive paint (Electrolube, UK) and secured with UV-curable adhesive (Dymax, CT, USA).

**Table 1:** Coordinates of brain regions targeted by recording implant

Target brain region	Center coordinates (mm)	Main references
Rostral forelimb area (RFA)	AP 3.75, ML ± 2.0, DV − 1.0	[60, 67]
Forelimb are of the primary motor cortex (M1FL)	AP 1.76, ML ± 2.71, DV − 1.0	[60, 68]
Trunk area of the primary motor cortex (M1Tr)	AP − 0.75, ML ± 1.625, DV − 1.0	[60]
Dorsomedial striatum (DMS)	AP 0.11, ML ± 2.65, DV − 3.75	[69]
Dorsolateral striatum (DLS)	AP 0.11, ML ± 4.07, DV − 4.0	[69]
Globus pallidus pars externa (GPe)	AP − 1.0, ML ± 3.125, DV − 6.125	[70]
Substantia nigra pars reticulata (SNr)	AP − 5.5, ML ± 2.45, DV − 7.8	[71]

Center coordinates are given in mm relative to bregma (for AP and ML) and cortical surface (for DV) with tooth bar in flat skull position

### Implantation Surgery

Electrode implantation surgeries were performed at least 6 weeks after 6-OHDA lesions (Fig. 1A). Animals were anaesthetised with fentanyl/medetomidine (0.21/0.21 mg/kg, i.p.; Apoteket AB, Sweden) and fixed in a stereotaxic frame (David Kopf Instruments, CA, USA) in a flat skull position. Holes were drilled in the frontal and parietal skull bone over the target recording sites followed by durotomy to accommodate a bilateral implantation of the microwire arrays. Silver wire was attached to five screws in the frontal and occipital skull bone for ground connection. The implant was anchored to the skull screws with dental acrylic cement (Kerr, CA, USA) also covering the ground wires for electrical insulation. After surgery, the anaesthesia was reversed by atipamezole hydrochloride (0.5 mg/kg, i.p.; Apoteket AB, Sweden), and buprenorphine (0.05 mg/kg, s.c.; Apoteket AB, Sweden) was administered as postoperative analgesic. The animals were allowed to recover for a minimum of 1 week before recording sessions commenced. During this period, they received extra postoperative care until fully recovered.

### Electrophysiological Recording Sessions

For one animal, a flat signal was obtained in all recordings and it was excluded from the electrophysiological analysis. In total, 79 recordings from 10 unilaterally 6-OHDA-lesioned dyskinetic rats were included in the study, of which 7 animals completed the entire series of treatments. During recording sessions, animals were placed individually in a transparent plastic cylinder (diameter 55 cm, height 40 cm) and their behaviour was recorded with digital video from the side and above (25 frames per second, Genie HM640 GigE camera; Teledyne DALSA, Canada) in parallel with electrophysiological signals (see the “Signal Acquisition” section). Synchronisation between video frames and electrophysiological signals was performed using a Master-8 pulse generator (A.M.P.I, Israel).

All recording sessions followed the same timeline. After being placed in the cylinder, animals were allowed to habituate for 10 min before they were briefly anaesthetised with isoflurane (5%) to connect custom-build adapters, Intan recording headstages (Intan Technologies, LLC, California, CA, USA), and recording cables to the implant. After recovering from the anaesthesia, animals were recorded for 30 min to establish baseline conditions and then injected with vehicle. After 20 min of recording, animals received an injection of either L-DOPA, SKF82958, sumanirole, or vehicle followed by a further 3 h of recording before ending the experiment. For details on the pharmacological paradigm see the “Drug Treatments” section.

### Signal Acquisition

Wideband neuronal activity was recorded with the Open Ephys acquisition system [41] using four Intan RHD2132 amplifier chips with on-board AD converters (Intan Technologies, LLC, California, CA, USA). The wideband signals were bandpass filtered between 1 and 7600 Hz and digitised and recorded at 30 kHz. Then, LFPs were extracted offline, low-pass filtered (8th order Butterworth at 500 Hz) and downsampled to 2000 Hz.

### Time–Frequency Analysis of LFP Power

**Fig. 2:**
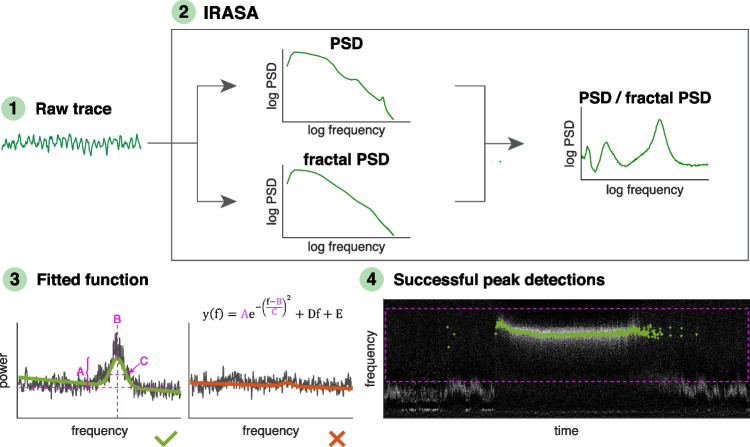
Representation of the method used to detect LFP oscillations. The analysis proceeded through the following steps: (1) Time series of power spectral densities (PSDs) were estimated for each bipolar LFP time series. (2) These mixed times series (composed of both fractal and oscillatory signals) were used as input for irregular-resampling auto-spectral analysis (IRASA). By irregularly resampling the time series multiple times, the fractal components could be separated from the mixed time series (left). The PSD was the normalised to the fractal component to construct a power spectrum measure that emphasises truly rhythmic activity (right). (3) To detect oscillations in each frequency band of interest, we fitted a parametric model to the spectral peak. Examples of LFP power spectra (grey) from 8 s of data are shown and green or red lines show the corresponding fitted function y(f). Functions with parameter values and goodness-of-fit within given limits were counted as successful peak detections and marked in green. (4) Example of a spectrogram where each green point marks an 8-s bin with successful peak detection. Hatched pink square marks the segment of spectrum fitted to only detect the frequency band of interest. Examples for each treatment can be found in Supplemental Fig. S3

To emphasise the local sources of the measured electrical potential, bipolar LFP time series from all unique pairs of wires within the same structure were computed offline for each LFP recording using custom code written in MATLAB (MathWorks). For each of these time series, a spectrogram (i.e. time series of power spectral densities (PSDs)) was estimated over the 0–250 Hz frequency range with Welch’s method (8 s Hann window with 50% overlap). To separate oscillatory components in the power spectrum from the arrhythmic, so-called fractal components, we used the irregular-resampling auto-spectral analysis (Wen and Liu [42]) (Fig. 2). By irregularly resampling the time series multiple times and then normalising the spectrum to the fractal component, it was possible to construct a power spectrum measure that emphasises truly rhythmic activity:

$${\mathrm{S}}_{\mathrm{dB}\left(\mathrm{fractal}\right)}\left(\mathrm{f}\right)=10{\mathrm{log}}_{10}\frac{\mathrm{S}(\mathrm{f})}{{\mathrm{S}}_{\mathrm{fractal}}(\mathrm{f})}$$

where S(f) and S_fractal_(f) have the dimension power per frequency (i.e.V^2^/Hz), and S_dB(fractal)_ is expressed in the dimensionless unit dB_fractal_. As a final step, spectrograms from each individual local bipolar LFP time series were averaged to create one representative spectrogram from each structure. Channels with exceptional noise levels were excluded based on visual inspection. Also, power line noise (50 ± 2 Hz and harmonics at 100 ± 1 Hz, 150 ± 1 Hz and 200 ± 1) was removed from the PSDs.

### Detection of Oscillatory Activity

To detect oscillations in the theta, beta, and NBG bands, we defined a parametric model

$$\mathrm{y}\left(\mathrm{f}\right)={\mathrm{Ae}}^{-{\left(\frac{\mathrm{f}-\mathrm{B}}{\mathrm{C}}\right)}^{2}}+\mathrm{Df}+\mathrm{E}$$

and fitted it to the spectral peak (Fig. 2). The parameters *A* (peak height), *B* (peak frequency), *C* (peak width), *D* (inclination of flat background), and* E* (offset of flat background) were estimated such that the model y(f) fit the spectrum optimally in the least-squares sense. The reliability of the estimated model was assessed by the goodness of fit (R2). Since the model only included one peak, different segments of the spectrum were fitted depending on the frequency band of interest: [1 15] Hz for theta, [8 48] Hz for beta, and [40 120] Hz for NBG (Fig. 2). Limit values for the fitted parameters and R2 were defined through visual inspection, and if the parameters for a given spectrum fell inside those limits, a positive detection was declared (Fig. 2; see Supplemental Table S1 for typical limit values).

This procedure resulted in a possible detection each 4 s. The detection rate was then calculated as the average of the binary outcome for each treatment within the structures of interest. If sufficient detections were made (> 5%), further analysis of the oscillation intensity (the height of the peak) and oscillation frequency was performed.

### Phase Synchrony and Functional Connectivity

Phase coherence was investigated on simultaneously recorded NBG signals from all structures in which these oscillations were detected during peak dyskinesia, and for all treatments. Signals were initially bandpass-filtered $\pm$ 5 Hz around the median frequency. Then, the Hilbert transform of each signal was computed with the MATLAB function *hilbert* (MathWorks). The instantaneous phase angle $\varphi \left(t\right)$ was then obtained by calculating $\varphi \left(t\right)= \mathrm{arg}z(t)$ using the MATLAB function *angle* (MathWorks). The mean phase difference between a given pair of wires *i* and *j* was calculated as $\Delta {\varphi }_{ij}=\langle {\varphi }_{i}\left(t\right)-{\varphi }_{j}\left(t\right)\rangle$, where $\langle \rangle$ denotes the circular mean (*circ_mean*, MATLAB CircStat toolbox; [43]). Similarly, the resultant vector length ${r}_{ij}$ was obtained using the function *circ_r* of the same toolbox.

To obtain a measure of phase coherence between two structures, ${r}_{ij}$ for all unique pairs with wire *i* in the first structure and wire *j* in the second structure were averaged. Similarly, the mean phase difference between two structures was obtained by averaging all unique $\Delta {\varphi }_{ij}$. However, to obtain a good quality phase estimate, we only included $\Delta {\varphi }_{ij}$ for pairs with a mean resultant vector length > 0.5, assuming that a higher magnitude reflects a higher signal to noise ratio of the source of interest and thus a better-quality phase estimate.

### Video Tracking Analysis

The position of each rat was tracked from the top camera video recordings with the open-source tool DeepLabCut [44]. Briefly, a total of 1221 frames picked randomly from 23 videos with different brightness conditions were used to label different body parts of the rat in order to train a deep learning network over 1,030,000 iterations. The network was then fed with all top camera videos from this study to track the chosen body parts.

Next, x and y coordinates of 7 body parts, that is, nose tip, left and right ear, head top (middle point on the implant between both medial headstages), body center (point on the midline running on the back between the thorax and abdomen), base of tail, and tip of tail, were extracted using MATLAB (MathWorks), considering only a detection likelihood of > 0.9. The centroid of the animal was calculated by averaging the x and y coordinates of all 7 body parts and the front of the animal by averaging x and y coordinates from nose, left and right ear, and head top. The direction of the animal was defined as the direction of the line between the front of the animal and the base of the tail.

To reveal the egocentric rotational behaviour of the rat, the number of full 360° turns was counted as follows: The direction measurements were binned into 8 segments. If the direction measurements passed through all 8 sectors in the correct order, it was counted as a full turn. Contralateral and ipsilateral turns were counted separately. An incomplete rotation reset the counting of sector transitions and the last angle in the discarded rotation was used as the first angle of the next count. The start and end frames of each full turn were used to determine the distance travelled during the turn and the duration of the turn.

Overall motor activity was computed by dividing the recording chamber into 1 × 1 cm squares and counting the number of squares visited in 1 min recording bins with reference to the body centroid. The number of squares visited was then divided by the total number of squares, and the corresponding value was represented as “fraction of total area visited”. In addition, the speed of overall motor activity was calculated based on the translation of the body centroid during a 1 s window. *Overall motor activity*, *overall motions*, and *open field motions* are used interchangeably to describe motor activity in the open field arena.

### Computed Tomography (CT) Scanning of the Implanted Microwire Arrays

The locations of recording wires were identified using computed tomography (CT) scanning of the heads with the microwire arrays still implanted. CT scans were performed on a Mediso NanoScan PET/CT scanner (Mediso, Hungary). To minimise CT metal-artefacts, the animal head was positioned such that the wires were perpendicular to the photon beam. This was achieved by placing the head on its side inside the tunnel such that the tunnel axis was aligned with the DV direction. The CT was performed as a helical scan with 0.30 pitch, 700 projections with 300 ms exposure time, 70 kV tube voltage, and 72 µA tube current. Images were reconstructed with thin-slice Ram-Lak filter, to give a 30 µm side, and 34 µm slice thickness (voxel size). The tip of each wire was manually identified in the resulting images. Bregma and lambda were used as landmarks to register the images to an anatomical atlas of the rat brain [45], followed by a manual calibration to visually optimise the alignment between atlas and scan. The atlas coordinates of the wire tips were calculated from the voxel coordinates using the resulting affine transformation. Finally, each wire was assigned an appropriate anatomical label based on the location of the wire tip in the atlas (Fig. 1D–E).

### Histological Verifications

Animals were anaesthetised with a lethal dose of sodium pentobarbital (100 mg/kg i.p., Apoteksbolaget AB, Sweden) and transcardially perfused with 0.9% saline followed by 4% paraformaldehyde before decapitation. Heads were kept in paraformaldehyde at 4 °C for post-fixation. After CT scanning of the head for electrode placement verification (see the “Computed Tomography (CT) Scanning of the Implanted Microwire Arrays” section), the brains were extracted and transferred to 25% sucrose solution in 0.1 M phosphate buffer (PB) at 4 °C until sinking for cryoprotection (at least 1 day). Coronal sections of 30 µm were cut serially through the entire brain using a microtome and stored at − 20 °C in non-freezing buffer solution until further processing for immunohistochemistry. The extent of dopamine denervation was verified in each animal by immunohistochemical staining for tyrosine hydroxylase (TH) (see Supplemental Methods). The placement of electrodes was evaluated in cresyl violet-stained sections by microscopic analysis and compared to CT scans of the implants to verify the locations of recording wires (Fig. 1E). Wires that did not target the structures of interest were excluded from further analysis.

### Statistical Analysis

Statistical analysis of behavioural data was performed using Prism 9 (GraphPad Software). Comparisons of treatment effects between groups, on single sessions or time points, were done with a mixed-effects model with Geisser-Greenhouse correction, followed by post hoc Tukey’s multiple comparisons test. All behavioural data compared between groups on single sessions or time points are presented as box plot and median, with whiskers annotating minimum and maximum values.

Detection rates of LFP frequency bands were compared with a generalised linear mixed-effects model (R *glmer* function in lme4 package) with binomial distribution and 4 factors (*treatment*, *structure*, *recording*, and *animal*). The factors *recording* and *animal* were considered random with *recording* nested in *animal*. *Hemisphere* was included as factor when appropriate. Bias adjustment was applied to account for the independent sources of random variation estimated by the model. Scheffé’s multiple comparisons post hoc test was chosen and results are reported as odds ratios (OR) (details on multiple comparisons can be found in Supplemental statistical materials—Detections).

Comparisons of peak height (absolute power) and peak frequency were done with linear mixed-effects models (R *lmer* function in lme4 package) with log-normal distribution when appropriate and factors *treatment*, *structure*, *recording*, and *animal*. The factors *recording* and *animal* were considered random with *recording* nested in *animal*. Scheffé’s multiple comparisons post hoc test was chosen and results are reported as estimates of differences or ratios (details on multiple comparisons can be found in Supplemental statistical materials – Peak height or Supplemental statistical materials – Peak frequency).

Comparisons of instantaneous phase differences were done with a MANOVA approach based on trigonometric functions of the circular data [46] using RStudio with factors *treatment* and *structure.* In addition, a one sample test for the mean angle was applied (*circ_mtest* in MATLAB CircStat toolbox [43]). Scheffé’s multiple comparisons post hoc test was chosen and results are reported as estimates of differences. Comparisons of phase coherence were done with Wilcoxon signed-rank test (MATLAB *signrank* function) (details on multiple comparisons can be found in Supplemental statistical materials – Phase or Supplemental statistical materials—Functional connectivity).

All electrophysiological parameters are given in mean $\pm$ SEM unless otherwise stated. Relations between variables were examined using the Spearman’s Rho. The level of statistical significance was set at α = 0.05 and *p*-values for detection rates, peak height, and peak frequency were adjusted to obtain multiplicity-adjusted results. To account for multiple testing in phase analysis, the Bonferroni correction was applied setting the significance at α/n.

## Results

### Patterns of Dyskinesia and Rotational Behaviour Induced by D1 vs. D2 Receptor Stimulation

All 6-OHDA-lesioned rats in this study were primed with daily L-DOPA treatment at the dose of 6 mg/kg/day, which corresponds to a therapeutic-like dose for this PD model [47–49]. After implanting the recording electrodes, all animals were treated sequentially with L-DOPA, followed by 2.0 mg/kg sumanirole (D2 receptor agonist), and then 0.05 mg/kg SKF82958 (D1 receptor agonist) over a period of several weeks (Fig. 1A). The doses of sumanirole and SKF82958 were carefully selected based on pilot experiments in non-implanted rats (Supplemental Fig. S2) as well as previous literature [29–34]. These doses represent a mid-dose range, where off target effects of the selected compounds can be assumed to be negligible.

**Fig. 3:**
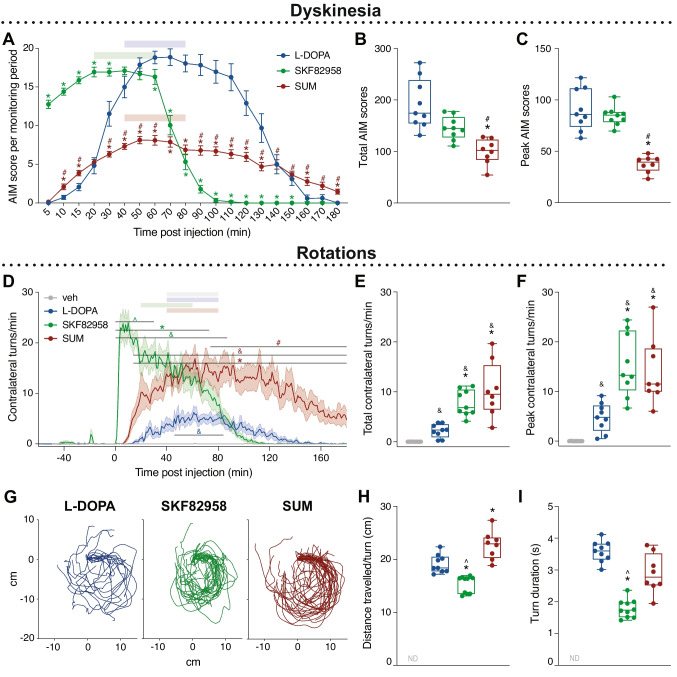
Effects of the D1R agonist, SKF82958, and the D2R agonist, sumanirole on inducing abnormal involuntary movements (AIMs) and rotations in comparison to L-DOPA in 6-OHDA lesioned rats. At time = 0 min, animals were injected with veh (grey, *n* = 8), L-DOPA (blue, *n* = 9), the D1R agonist SKF82958 (green, *n* = 9), or the D2R agonist SUM (red, *n* = 8). **A–C** Dyskinesia profiles. **A** Time course of global AIM scores (Mixed-effects model: F(treatment)_1.626,40.65_ = 10.35, *p* = 0.001; F(time)_4.593,114.8_ = 133.8, *p* < 0.001; F(interaction)_5.693,117.1_ = 73.01, *p* < 0.001). Coloured bars mark time period of peak dyskinesia chosen for further analysis. **B** Total AIM scores calculated as sum of global AIMs from 0 to 180 min post injection (Mixed-effects model: F(treatment)_1.279,14.71_ = 15.49, *p* = 0.001). **C** Peak AIM scores calculated as sum of global AIMs during peak dyskinesia (cf. coloured bars in A) (Mixed-effects model: F(treatment)_1.362,10.21_ = 40.36, *p* < 0.001). **D–I** Rotational behaviour. **D** Time course of completed contralateral rotations per min. Coloured bars mark peak time period chosen for further analysis (Mixed-effects model: F(treatment)_3,6805.9_ = 1578.9, *p* < 0.001; F(time)_240,6799.1_ = 22.03, *p* < 0.001; F(interaction)_694,6799_ = 12.26, *p* < 0.001). **E** Completed contralateral rotations calculated as mean from 0 to 180 min post injection (Mixed-effects model: F(treatment)_1.510,10.57_ = 21.89, *p* < 0.001). **F** Completed contralateral rotations calculated as mean during peak time period (cf. coloured bars in D) (Mixed-effects model: F(treatment)_1.838,12.86_ = 19.79, *p* < 0.001). **G** Aligned traces representative of the turning characteristics within 1 min recording at the peak of drug effect (SKF82958: 40–41 min; L-DOPA, SUM: 60–61 min) for one recording per treatment. **H** Distance travelled in cm per turn during peak time period (cf. coloured bars in D) (Mixed-effects model: F(treatment)_1.838,13.79_ = 34.31, *p* < 0.001). **I** Duration in s per turn during peak time period (cf. coloured bars in D) (Mixed-effects model: F(treatment)_1.518.18.22_ = 43.85, *p* < 0.001). Tukey’s post hoc: &*p* < 0.05 vs. veh, *p < 0.05 vs. L-DOPA, #*p* < 0.05 vs. SKF82958, ^*p* < 0.05 vs. SUM

Dyskinetic behaviours were induced by all the compounds tested, although with differences in time course and overall severity (Fig. 3A–C). L-DOPA induced dyskinesia in all animals, reaching peak severity at 50–60 min post injection (Fig. 3A), with AIM scores comparable to those previously reported from non-implanted rats [48, 49]. SKF82958 resulted in an immediate induction of dyskinesia yielding a severity peak at 20–30 min post injection, though with AIM scores similar to those recorded during the severity peak of L-DOPA (Fig. 3A, C). However, the time course was significantly shorter compared with both L-DOPA and sumanirole, and AIM scores showed an abrupt decrease down to baseline between 70 and 100 min post injection (Fig. 3A). After treatment with sumanirole, both total and peak AIM scores were significantly lower compared to both L-DOPA and SKF82958 (Fig. 3B–C; Total: − 41% vs L-DOPA, *p = 0.008; − 30%, vs. SKF82958, #*p* = 0.004; Peak: − 54% vs. L-DOPA, *p = 0.001; − 54% vs SKF82958 #*p* < 0.001). Similar to L-DOPA, peak AIM severity with sumanirole was seen at 50–60 min post injection (Fig. 3A). In order to compare the treatments during functionally analogous time windows, we focused our analyses on the periods corresponding to peak AIM severity, i.e., 20–60 min post injection for SKF82958 and 40–80 min for both L-DOPA and sumanirole (see coloured bars in Fig. 3A).

Analysis of the individual AIM subtypes revealed that axial, limb, and orolingual scores were all significantly lower after sumanirole treatment compared with L-DOPA and SKF82958 (Supplemental Fig. S4A-C, E–G). Significant differences between the agonists and L-DOPA were detected upon expressing each AIM subtype scores as a percentage of the total scores recorded from each animal during the test session. Compared with L-DOPA, sumanirole treatment induced a higher relative expression of axial AIMs and lower expression of orolingual AIMs (Supplemental Fig. S4D, H).

Investigating rotational behaviour, we found a difference in the time course between treatments (Fig. 3D). Indeed, both dopamine agonists induced more contralateral turns compared to L-DOPA (Fig. 3E-F; Total: *p = 0.002 SKF82958 vs. L-DOPA, *p = 0.012 SUM vs. L-DOPA; Peak: *p = 0.012 SKF82958 vs. L-DOPA, *p = 0.019 SUM vs. L-DOPA), whereas no ipsilateral turns were detected after any treatment (data not shown). The dynamics of the turns were different between the dopamine agonists (Fig. 3G). Characterising single turns during peak time revealed that sumanirole induced wide and slow turns, whereas turns induced by SKF82958 were narrow and fast (Fig. 3H: *p = 0.002 SKF82958 vs. L-DOPA, *p = 0.023 SUM vs. L-DOPA, ^*p* < 0.001 SKF82958 vs. SUM; Fig. 3I: *p < 0.001 SKF82958 vs. L-DOPA, ^*p* = 0.006 SKF82958 vs. SUM). In agreement with these results, SKF82958 resulted in smaller overall motions in the test arena (Supplemental Fig. S4I-L).

Taken together, these results show that dyskinetic behaviours developed upon pharmacological stimulation of both D1 and D2 receptors in L-DOPA-primed animals, although dyskinesia severity was milder upon treatment with the D2 agonist compared to both L-DOPA and SKF82958. In addition, both dopamine agonists induced more contralateral turns compared to L-DOPA, these turns being faster upon D1 receptor stimulation.

### Dyskinetic Behaviours Concur with Network-Wide Changes in LFP Activity Patterns

**Fig. 4:**
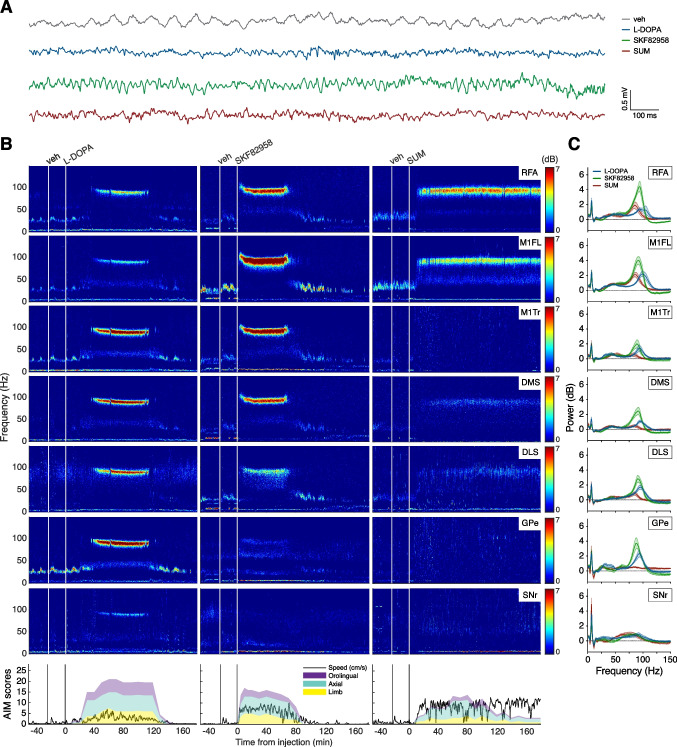
Spectral analysis of multi-structure local field potential (LFP) recordings. **A **Representative examples of raw LFP traces recorded in M1FL of the lesioned hemisphere following vehicle treatment (grey) or during peak dyskinesia following treatment with L-DOPA (blue), the D1R agonist SKF82958 (green), or the D2R agonist SUM (red). **B **Representative spectrograms from one recording per treatment represent the time–frequency plots of LFP spectral power in cortico-basal ganglia structures of the lesioned hemisphere following treatment with L-DOPA (left column), SKF82958 (middle column), or SUM (right column). White lines mark various drug injections. In the bottom panel, the time course of the corresponding behaviours is presented as the severity of three subtypes of dyskinetic motor symptoms (axial, limb, and orolingual AIMs) and the speed of open field motions (cm/s). Black lines mark various drug injections. **C **Averaged LFP power spectra (0–150 Hz) in cortico-basal ganglia structures of the lesioned hemisphere during peak dyskinesia (cf. Figure 3A) following treatment with L-DOPA (blue, *n* = 9), SKF82958 (green, *n* = 9), and SUM (red, *n* = 8). Abbreviations: rostral forelimb area (RFA), primary motor cortex forelimb area (M1FL), primary motor cortex trunk area (M1Tr), dorsomedial striatum (DMS), dorsolateral striatum (DLS), globus pallidus pars externa (GPe), and substantia nigra pars reticulata (SNr)

In parallel with the behavioural assessment, LFP recordings were performed simultaneously in 7 nodes of the cortico-basal ganglia network of both the intact and the lesioned hemisphere. Indeed, we recorded from the rostral forelimb area (RFA), two primary motor cortical areas suggested to be involved in forelimb (M1FL) and trunk (M1Tr) movements, the dorsolateral (DLS) and dorsomedial (DMS) striatum, and the primary targets of striatal projection systems, that is the globus pallidus pars externa (GPe) and the substantia nigra pars reticulata (SNr) (Table 1). An example multi-site recording for each treatment is illustrated in Fig. 4B with the time course of the corresponding AIM scores and speed of open field motions presented in the bottom panel. We noted a close association between the expression of dyskinetic behaviours and the occurrence of NBG LFP oscillations (70–110 Hz) in several of the recorded structures in the lesioned hemisphere. Spectral contents were in fact altered in several frequency bands during peak-of-dose dyskinesia (Fig. 4B). In particular, beta oscillations (12–35 Hz) were generally decreased compared to vehicle and baseline conditions (see Fig. 4A and the interval preceding drug injection in Fig. 4B). The occurrence of beta oscillations in PD has been covered extensively elsewhere [3–7, 12, 19, 50–53], and has furthermore shown little association to dyskinesia [19]. Therefore, beta oscillations will not be investigated further in this study. In addition to NBG oscillations, several of the recorded structures exhibited a prominent increase in theta oscillations (5–10 Hz). A summary of the LFP power spectra during peak-of-dose dyskinesia is shown in Fig. 4C, which indicates conspicuous increases in both the NBG (70–110 Hz) and the theta range (5–10 Hz) in most of the recorded structures.

In the following, we will therefore focus our analyses on these frequency bands and compare the effects of different treatments during peak AIM severity, i.e. 20–60 min post injection for SKF82958, 40–80 min for L-DOPA, sumanirole, and vehicle (cf. coloured bars in Fig. 3D).

### Differential Effects of D1 and D2 Receptor Agonists on Narrowband Gamma (NBG) Oscillations (70–110 Hz)

**Fig. 5:**
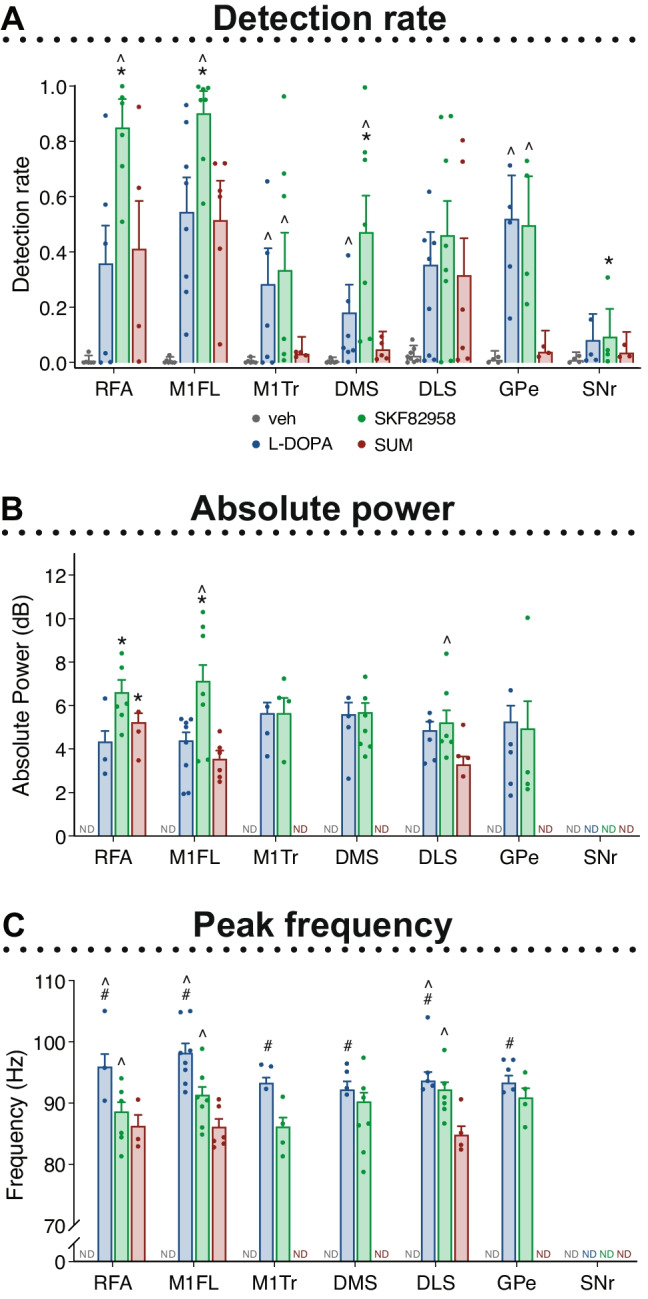
Effects of treatments on cortico-basal ganglia NBG oscillations in the lesioned hemisphere during peak dyskinesia. Within each structure, effects of L-DOPA (blue), the D1R agonist SKF82958 (green), and the D2R agonist SUM (red) were compared. **A **Detection rate of NBG oscillations (Mixed-effects model: χ^2^(treatment)_2_ = 22.686, *p* < 0.001; χ^2^(structure)_6_ = 32,792, *p* < 0.001; χ.^2^(interaction)_12_ = 6939.1, *p* < 0.001). **B **Absolute power (dB) of the detected peaks (Mixed-effects model: F(treatment)_2,50_ = 6.47, *p* = 0.003; F(structure)_6,64268_ = 561.9, *p* < 0.001; F(interaction)_8,64283_ = 1386.6, *p* < 0.001). **C **Frequency (Hz) of the detected peaks (Mixed-effects model: F(treatment)_2,48_ = 94.63, *p* < 0.001; F(structure)_6,64256_ = 145.6, *p* < 0.001; F(interaction)_8,64269_ = 66.3, *p* < 0.001). Dots mark the mean per animal. Scheffe’s post hoc: *p < 0.05 vs. L-DOPA, #*p* < 0.05 vs. SKF82958, ^*p* < 0.05 vs. SUM. Effects of treatments on NBG oscillations in the intact hemisphere can be found in Supplemental Fig. S6. Abbreviations: rostral forelimb area (RFA), primary motor cortex forelimb area (M1FL), primary motor cortex trunk area (M1Tr), dorsomedial striatum (DMS), dorsolateral striatum (DLS), globus pallidus pars externa (GPe), and substantia nigra pars reticulata (SNr)

Following treatment with L-DOPA and the dopamine agonists, NBG oscillations appeared in several of the recorded structures of the lesioned hemisphere coinciding temporally with the AIMs (Fig. 4B–C and Supplemental Fig. S5A), which is in line with previous findings in animal models of LID and reports from dyskinetic PD patients [12, 16, 18, 19, 21]. NBG oscillations were not detected in the intact hemisphere, nor were they detected after vehicle treatment in either hemisphere (Fig. 5A and Supplemental Fig. S6). The below report is therefore focused on a comparison between the three pharmacological treatments in the lesioned hemisphere.

In all the recorded cortical structures, the detection rate of NBG oscillations was increased significantly by SKF82958 compared to all other treatments, with a stronger effect in the RFA and M1FL areas than in M1Tr (Fig. 5A; RFA: OR_L-DOPA/SKF82958_ = 0.17 $\pm$ 0.04, *p < 0.001; OR_SKF82958/SUM_ = 17.5 $\pm$ 6, ^*p* = 0.009; M1FL: OR_L-DOPA/SKF82958_ = 0.38 $\pm$ 0.09, *p < 0.001; OR_SKF82958/SUM_ = 22.6 $\pm$ 7.7, ^*p* = 0.001; M1Tr: OR_SKF82958/SUM_ = 130 $\pm$ 45.3, ^*p* < 0.001). Sumanirole and L-DOPA induced similar detection rates in RFA and M1FL, whereas in the M1Tr, NBG oscillations induced by sumanirole were below the threshold for detection (see the “Detection of Oscillatory Activity” section) (Fig. 5A; M1Tr: OR_L-DOPA/SUM_ = 52.9 $\pm$ 14.9, ^*p* < 0.001). Indeed, following sumanirole treatment, the NBG detection rate was below the threshold not only in M1Tr, but also in DMS, GPe, and SNr. Accordingly, L-DOPA and SKF82958 induced higher detection rates compared to sumanirole both in the DMS and GPe (Fig. 5A; DMS: OR_SKF82958/SUM_ = 131 $\pm$ 45.2, ^*p* < 0.001; OR_L-DOPA/SUM_ = 11.9 $\pm$ 3.3, ^*p* = 0.015; GPe: OR_SKF82958/SUM_ = 126 $\pm$ 44.1, ^*p* < 0.001; OR_L-DOPA/SUM_ = 112 $\pm$ 32, ^*p* < 0.001). In the DMS, the detection rate was moreover significantly higher after SKF82958 treatment compared to L-DOPA (Fig. 5A; DMS: OR_L-DOPA/SKF82958_ = 0.25 $\pm$ 0.06, *p < 0.001), whereas no differences between treatments were found in the DLS (Fig. 5A). In the SNr, the detection rate of NBG oscillations was below the threshold for all treatments. Thus, there was an increased detection rate after treatment with SKF82958 in most structures of the lesioned hemisphere compared to the other two treatments. In contrast, the detection rate for sumanirole was lower, and NBG was not detectable in several of the recorded structures (M1Tr, DMS, GP, and SNr).

When the NBG oscillations were detected (see the “Detection of Oscillatory Activity” section), a further analysis of the power and frequency of the detected peaks was performed. The NBG power of the detected peaks was increased by SKF82958 in several of the recorded structures (Fig. 5B). In forelimb motor cortices (RFA and M1FL), treatment with SKF82958 increased NBG power markedly above L-DOPA and sumanirole (Fig. 5B; RFA: ratio_L-DOPA/SKF82958_ = 0.65 $\pm$ 0.04, *p = 0.001; M1FL: ratio_L-DOPA/SKF82958_ = 0.57 $\pm$ 0.04, *p < 0.001; ratio_SKF82958/SUM_ = 2.03 $\pm$ 0.13, ^*p* < 0.001). In addition, NBG power in the RFA was increased after sumanirole treatment compared to L-DOPA (Fig. 5B; RFA: ratio_L-DOPA/SUM_ = 0.69 $\pm$ 0.05, *p = 0.02). In the DLS, there was a ~ 50% increase in NBG power after treatment with SKF82958 compared to sumanirole (Fig. 5B; DLS: ratio_SKF82958/SUM_ = 1.47 $\pm$ 0.1, ^*p* = 0.003). In the M1Tr, DMS, and GPe, NBG power was comparable upon treatment with SKF82958 and L-DOPA (sumanirole did not induce NBG oscillations in these regions) (Fig. 5B).

The NBG oscillation frequencies were clearly modulated by the pharmacological treatments, but displayed very small differences between structures during peak dyskinesia (Fig. 5C and Supplemental Table S5). These oscillations had a mean frequency of 94.9 $\pm$ 6.1 Hz across structures after treatment with L-DOPA, 90.1 $\pm$ 5.4 Hz after SKF82958, and 85.7 $\pm$ 4.9 Hz after sumanirole treatment. When comparing the treatments within each structure, the frequency of the detected NBG oscillations was higher after L-DOPA treatment compared to both SKF82958 and sumanirole in all structures (Fig. 5C, and Supplemental Table S5; RFA, M1FL, and DLS: #/^*p* < 0.001 L-DOPA vs. SKF82958 and SUM; M1Tr, DMS, and SNr: #*p* < 0.001 L-DOPA vs. SKF82958). Additionally, the frequency of the detected peaks was higher after SKF82958 treatment compared to sumanirole in all structures (Fig. 5C and Supplemental Table S5; RFA, M1FL, and DLS: ^*p* < 0.001 SKF82958 vs. SUM).

To further emphasise the relation between LFP oscillations in the NBG range and dyskinesia, we calculated the correlation of NBG power against manually scored AIMs. Indeed, NBG oscillations were strongly correlated with AIM scores in the majority of structures recorded, being strongest for M1FL (Rho = 0.680, *p* < 0.001, Supplemental Fig. S8A). Significant positive correlations were moreover found between NBG power and each individual AIM subtypes (see Supplemental Table S2).

Because of the correlation found between NBG oscillations and dyskinesia severity, we examined this oscillatory activity at time points when the dopamine receptor agonists were producing equivalent AIM scores (i.e. 70–80 min after the administration of SKF82958/sumanirole). At these time points too, NBG detection rates in subcortical structures were significantly lower after sumanirole compared to SKF82958 administration (Supplemental Fig. S7A, B) and relative differences as to NBG power were maintained in most structures (Supplemental Fig. S7C). This suggests that the stronger action of D1 vs. D2 receptor stimulation in driving NBG oscillations is not contingent on an induction of more severe dyskinesia.

### D2 Receptor Stimulation Elicits Theta Oscillations (5–10 Hz) in the Deep Basal Ganglia Nuclei

**Fig. 6:**
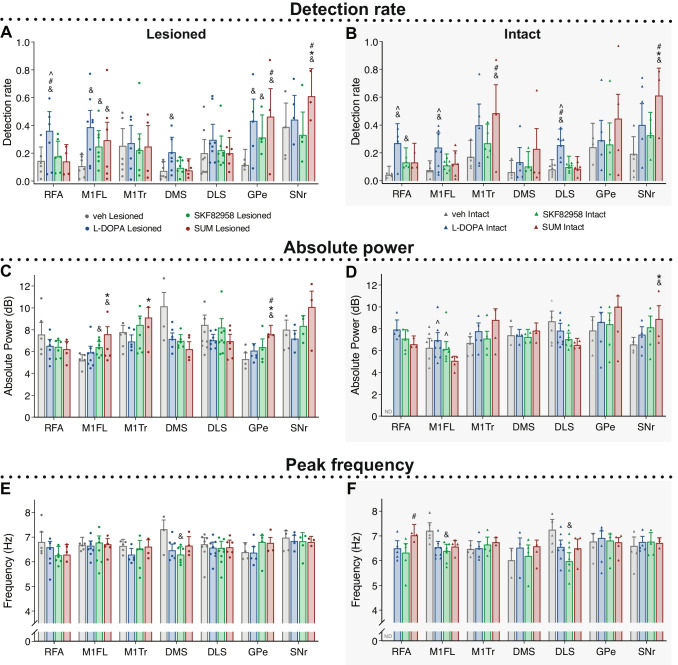
Effects of treatments on cortico-basal ganglia theta oscillations during peak dyskinesia. Within each structure, effects of L-DOPA (blue), the D1R agonist SKF82958 (green), the D2R agonist SUM (red), and veh treatment (grey) were compared in the lesioned (left column) and intact (grey shaded, right column) hemisphere. **A–B** Detection rate of theta oscillations in lesioned **A** and intact **B** hemisphere (Mixed-effects model(lesioned): χ^2^(treatment)_3_ = 29.715, *p* < 0.001; χ^2^(structure)_6_ = 8283.2, *p* < 0.001; χ^2^(interaction)_18_ = 3506.5, *p* < 0.001; Mixed-effects model(intact): χ^2^(treatment)_3_ = 31.738, *p* < 0.001; χ^2^(structure)_6_ = 10,998, *p* < 0.001; χ.^2^(interaction)_18_ = 4744.3, *p* < 0.001). **C–D** Absolute power (dB) of the detected peaks in lesioned **C** and intact **D** hemisphere (Mixed-effects model(lesioned): F(treatment)_3,64_ = 5.22, *p* < 0.001; F(structure)_6,48592_ = 4442.7, *p* < 0.001; F(interaction)_18,47513_ = 112.2, *p* < 0.001; Mixed-effects model(intact): F(treatment)_3,59_ = 0.47, *p* = 0.705; F(structure)_6,36858_ = 380.2, p < 0.001; F(interaction)_17,35699_ = 122.4, p < 0.001). **E–F** Frequency (Hz) of the detected peaks in lesioned **E** and intact **F** hemisphere (Mixed-effects model (lesioned): F(treatment)_3,62_ = 2.78, *p* = 0.048; F(structure)_6,48684_ = 19.69, *p* < 0.001; F(interaction)_18,47683_ = 37.8, *p* < 0.001; Mixed-effects model (intact): F(treatment)_3,63_ = 5.47, *p* = 0.002; F(structure)_6,37141_ = 29.9, *p* < 0.001; F(interaction)_17,36424_ = 45.7, *p* < 0.001). Dots mark the mean per animal. Scheffe’s post hoc: &*p* < 0.05 vs. veh, *p < 0.05 vs. L-DOPA, #*p* < 0.05 vs. SKF82958, ^*p* < 0.05 vs. SUM. Abbreviations: rostral forelimb area (RFA), primary motor cortex forelimb area (M1FL), primary motor cortex trunk area (M1Tr), dorsomedial striatum (DMS), dorsolateral striatum (DLS), globus pallidus pars externa (GPe), and substantia nigra pars reticulata (SNr)

Theta oscillations were present in all recorded structures in both the intact and lesioned hemispheres and had a mean frequency of 6.8 ± 1.2 Hz. The detection rate of theta oscillations in the lesioned hemisphere across structures was increased after treatment with vehicle, L-DOPA, and SKF82958 compared to the intact hemisphere, whereas sumanirole induced a bilateral increase in the detections of theta oscillations (Mixed-effects model: χ^2^(treatment)_3_ = 56.7, *p* < 0.001; χ^2^(hemisphere)_1_ = 733, *p* < 0.001; χ^2^(interaction)_3_ = 364, *p* < 0.001; OR_veh intact/veh lesioned_ = 0.63 $\pm$ 0.02, *p* < 0.001; OR_L-DOPA intact/L-DOPA lesioned_ = 0.80 $\pm$ 0.01, *p* < 0.001; OR_SKF82958 intact/SKF82958 lesioned_ = 0.84 $\pm$ 0.01, *p* < 0.001; OR_SUM intact/SUM lesioned_ = 1.1 $\pm$ 0.02, *p* = 1.0) (Fig. 6A–B). Furthermore, the detection rate of theta oscillations was constant during the time course of the dyskinetic behaviour with only small changes in the early and late phase of dyskinesia (Fig. 4B and Supplemental Fig. S5B).

When investigating the detection of theta oscillations within the cortical structures, the detection rate was increased in the M1FL area of the lesioned hemisphere by all treatments compared to vehicle (Fig. 6A; M1FL: OR_veh/L-DOPA_ = 0.21 $\pm$ 0.03, &*p* < 0.001; OR_veh/SKF82958_ = 0.36 $\pm$ 0.05, &*p* < 0.001; OR_veh/SUM_ = 0.26 $\pm$ 0.04, &*p* < 0.001). In addition, treatment with L-DOPA increased the detection rate of theta oscillations bilaterally in both RFA and M1FL as well as in the striatum compared to all other treatments (Fig. 6A–B and Supplemental Table S4; RFA_lesioned_: &/#/^*p* < 0.001 L-DOPA vs. all; DMS_lesioned_: &*p* < 0.001 L-DOPA vs. veh; RFA_intact_: &*p* < 0.001 L-DOPA vs. veh, ^*p* < 0.001 L-DOPA vs. SUM; M1FL_intact_: &*p* < 0.001 L-DOPA vs. veh, #*p* = 0.02 L-DOPA vs. SKF82958, ^*p* < 0.001 L-DOPA vs. SUM; DLS_intact_: &/#/*p* < 0.001 L-DOPA vs. all). In the GPe of the lesioned hemisphere, theta oscillations became more prevalent upon treatment with sumanirole compared to both SKF82958 and vehicle, and upon treatment with L-DOPA and SKF82958 compared to vehicle only (Fig. 6A; GPe: OR_veh/SUM_ = 0.15 $\pm$ 0.03, &*p* < 0.001; OR_SKF82958/SUM_ = 0.41 $\pm$ 0.06, #*p* = 0.001; OR_veh/L-DOPA_ = 0.33 $\pm$ 0.05, &*p* = 0.001; OR_veh/SKF82958_ = 0.43 $\pm$ 0.07, &*p* = 0.011). In the SNr, the detection rate of theta oscillations was increased bilaterally by sumanirole compared to all other treatments (Fig. 6A–B; SNr_lesioned_: OR_veh/SUM_ = 0.27 $\pm$ 0.05, &*p* < 0.001; OR_L-DOPA/SUM_ = 0.29 $\pm$ 0.05, *p < 0.001; OR_SKF82958/SUM_ = 0.22 $\pm$ 0.03, #*p* < 0.001; SNr_intact_: OR_veh/SUM_ = 0.14 $\pm$ 0.02, &*p* < 0.001; OR_L-DOPA/SUM_ = 0.26 $\pm$ 0.04, *p < 0.001; OR_SKF82958/SUM_ = 0.33 $\pm$ 0.05, #*p* < 0.001). Thus, there was an increased detection rate of theta oscillations after treatment with sumanirole in the deeper nuclei (GPe and SNr) compared to the other treatments, but the detection rate in the GPe was also increased after treatment with SKF82958 and L-DOPA compared to vehicle.

For the detected peaks (see the “Detection of Oscillatory Activity” section), the absolute power of theta LFPs in the motor cortex of the lesioned hemisphere was increased ~ 25% by sumanirole compared to both vehicle and L-DOPA, and a similar increase was seen in the M1FL area for SKF82958 compared to vehicle (Fig. 6C; M1FL_lesioned_: ratio_veh/SUM_ = 0.65 $\pm$ 0.03, &*p* < 0.001; ratio_L-DOPA/SUM_ = 0.75 $\pm$ 0.03, *p < 0.001; ratio_veh/SKF82958_ = 0.76 $\pm$ 0.03, &*p* = 0.007; M1Tr_lesioned_: ratio_L-DOPA/SUM_ = 0.75 $\pm$ 0.03, *p < 0.001). In comparison, the theta power of the M1FL area in the intact hemisphere was reduced by sumanirole compared to L-DOPA and SKF82958 (Fig. 6D; M1FL_intact_: ratio_L-DOPA/SUM_ = 1.46 $\pm$ 0.05, ^*p* < 0.001; ratio_SKF82958/SUM_ = 1.26 $\pm$ 0.04, ^*p* = 0.003). In the striatum, no differences were observed in the power of theta oscillations induced by the different treatments. This is in contrast to the GPe, where theta power was increased by ~ 20% in the lesioned hemisphere after treatment with sumanirole compared to all other treatments (Fig. 6C; GPe: ratio_veh/SUM_ = 0.72 $\pm$ 0.04, &*p* = 0.002; ratio_L-DOPA/SUM_ = 0.78 $\pm$ 0.03, *p = 0.001; ratio_SKF82958 /SUM_ = 0.79 $\pm$ 0.03, #*p* = 0.001). The absolute power of theta oscillations in the SNr tended to be higher with sumanirole compared to the other treatments (Fig. 6C; lesioned side: SNr: ratio_veh/SUM_ = 0.85 $\pm$ 0.04, *p* = 0.897; ratio_L-DOPA/SUM_ = 0.84 $\pm$ 0.03, *p* = 0.258; ratio_SKF82958 /SUM_ = 0.89 $\pm$ 0.03, *p* = 0.935), reaching statistical significance compared to both vehicle and L-DOPA (though not to SKF82958) in the intact hemisphere (Fig. 6D; intact side: SNr: ratio_veh/SUM_ = 0.74 $\pm$ 0.03, &*p* = 0.003; ratio_L-DOPA/SUM_ = 0.81 $\pm$ 0.03, *p = 0.019; ratio_SKF82958/SUM_ = 0.90 $\pm$ 0.03, *p* = 0.976). The oscillation frequencies recorded in different structures were substantially similar between treatments (Fig. 6E–F), with only few small differences found in the intact hemisphere (Fig. 6F).

To explore the relation between theta oscillations and dyskinesia, we calculated the correlation of theta power against global AIM scores. Weak, though significant, correlations were found in RFA, M1FL, and striatum (DMS and DLS) (Supplemental Fig. S8B). Noteworthy, a robust correlation between dyskinesia severity and theta power was found in the GPe of the lesioned hemisphere, where both the global AIM scores (Supplemental Fig. S8B; Rho = 0.338, *p* < 0.001) and the individual AIM subtypes (Supplemental Table S3) were significantly correlated with the power of theta oscillations.

Since increases in theta activity had also occurred on the intact side, we moreover analysed the correlations between theta power and an index of overall motions in the recording chamber (the fraction of total area visited). We focused this analysis on the GPe and SNr, where the largest increases in theta detection rate and power had been detected after treatment (see Fig. 6A–D). In both GPe and SNr, there was a positive correlation between the fraction of area visited and theta power in both the intact and lesioned hemisphere across treatments (Supplemental Fig. S9A, B). However, the relationship between overall motions and theta power became negative in the lesioned GPe specifically upon treatment with sumanirole (Supplemental Fig. S9A; Rho =  − 0.265, *p* = 0.002). This indicates that theta power measured in the dopamine-denervated GPe after sumanirole treatment was specifically correlated with the expression of dyskinesia, not with an increase in motor activity. In contrast, treatment-induced bilateral theta-increases in the SNr were not directly related to the presence of dyskinesia (cf. Supplemental Fig. S8B) but seemed to reflect an overall increase in motions (cf. Supplemental Fig. S9B).

### NBG Phase Synchrony Reveals a Stronger Effect of D1 Stimulation on Cortico-Subcortical Coupling

**Fig. 7:**
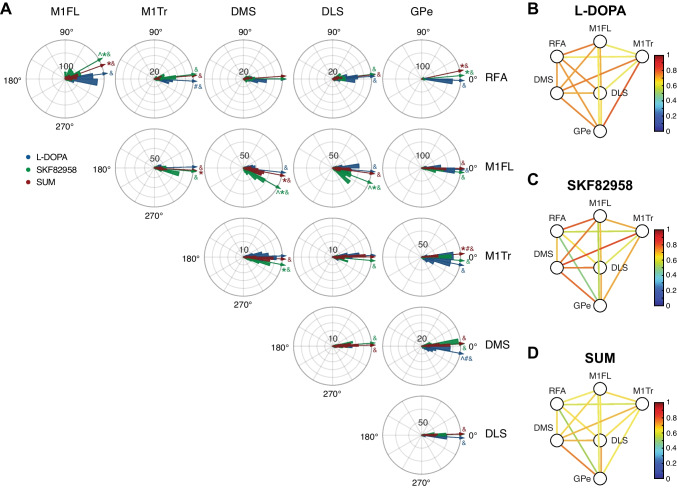
Phase synchrony and functional connectivity in the NBG frequency band. **A **Phase plots showing the distribution of instantaneous phase differences between pairs of structures for NBG oscillations. For distributions between 0 and 90° column structure leads row structure, whereas for distributions between 270 and 0° row structure leads column structure. Arrows reflect the mean angular direction of the distribution of the mean phase differences. Distributions are color-coded according to treatment (L-DOPA (blue), SKF82958 (green), and SUM (red)) (MANOVA: F(treatment)_2_ = 243, *p* < 0.001; F(structure)_27_ = 110, *p* < 0.001; F(interaction)_54_ = 26, *p* < 0.001). Scheffe’s post hoc: *p < 0.05 vs. L-DOPA, #*p* < 0.05 vs. SKF82958, ^*p* < 0.05 vs. SUM. Bonferroni correction: &*p* < 0.05 vs. 0°. **B–D **Diagrams of NBG functional connectivity for L-DOPA **B**, SKF82958 **C**, and sumanirole **D**, as represented by the magnitude of the resultant vector of the phase distributions (see colour scale). In the shown diagrams, all comparisons of functional connectivity between treatments are significant for each structure pair (Wilcoxon signed-rank test: z =  − 117, *p* < 0.001; see Supplemental Table S6). Abbreviations: rostral forelimb area (RFA), primary motor cortex forelimb area (M1FL), primary motor cortex trunk area (M1Tr), dorsomedial striatum (DMS), dorsolateral striatum (DLS), and globus pallidus pars externa (GPe)

To identify potential drivers of NBG oscillations in the cortico-basal ganglia network, we examined phase relationships between different structures during peak dyskinesia, as induced by L-DOPA, sumanirole, and SKF82958 (Fig. 7A). This revealed that the phase differences between NBG oscillations in different structures were not random, as would be expected if there was no functional coupling between the structures. Rather, we found a strong bias towards certain phase difference values. Using the strength of this bias as a measure of functional connectivity, we found a stronger functional coupling between structures in the lesioned hemisphere compared to the intact hemisphere (Supplemental Fig. S10). Notably, a tighter functional coupling was seen within cortex and between cortex and striatum in the lesioned hemisphere upon treatment with SKF82958 compared to sumanirole (Fig. 7C–D and Supplemental Table S6). While a tight functional coupling was found between striatum and GPe following all treatments, the GPe also showed strong functional connectivity with M1Tr and RFA after treatment with SKF82958/L-DOPA and L-DOPA, respectively (Fig. 7B–D and Supplemental Table S6).

Generally, NBG phase differences between structures were concentrated around 0°. However, several structure pairs showed a non-zero phase difference. As shown in Fig. 7A, M1FL was leading RFA and striatum for all treatments (Fig. 7A and Supplemental Table S7), with a differential effect depending on treatment. NBG oscillations in RFA were moreover lagging GPe for both SKF82958 and sumanirole, whereas L-DOPA-induced NBG oscillations in GPe were lagging M1Tr and DMS (Fig. 7A and Supplemental Table S7). For the remaining structures, NBG oscillations were phase synchronised.

Altogether, our data show that all recorded structures are strongly coupled with small phase differences < 30°, corresponding to delays < 1 ms. Also, the fact that M1FL leads RFA and striatum suggests that it might be an important driver of NBG oscillations.

## Discussion

Using in vivo multi-structure recordings in a rat model of LID, we have examined the pattern of cortico-basal ganglia oscillations induced by chronic treatment with potent and selective agonists of D1 and D2 receptors. The D1 agonist SKF82958 induced strong NBG oscillations in all recorded structures except for the SNr, whereas the D2 agonist sumanirole only induced NBG oscillations in forelimb motor cortical areas and dorsolateral striatum, and with significantly inferior power compared to SKF82958. In contrast, sumanirole had a stronger effect on the induction of theta oscillations, whose detection rate and power in the GPe and SNr were significantly larger than after SKF82958 treatment. To our knowledge, this is the first report on the effects of pharmacological D1 vs. D2 receptor stimulation on LFPs recorded simultaneously in many critical structures of the cortico-basal ganglia network. For the first time, we recorded simultaneously from motor cortical areas corresponding to forelimb (RFA and M1FL) or trunk (M1Tr) regions, from both associative and motor striatal regions (DMS and DLS, respectively), and from the primary targets of striatal projection systems that jointly control movement (GPe and SNr).

### Effects of D1 and D2 Receptor Agonists on NBG Activity

Previous studies in rat models of LID have shown that the expression of dyskinesia induced by L-DOPA coincides with the appearance of NBG oscillations in the motor cortex and several other nodes of the cortico-basal ganglia-thalamic network [15, 16, 18, 19, 53, 54]. Interestingly, NBG oscillations have been described in the motor cortex and subthalamic nucleus of dyskinetic PD patients [21], and have been proposed as a promising control signal for adaptive DBS [22]. Of note, this NBG oscillation was minimally affected by voluntary movements, while its presence was coinciding with dyskinesia [21], suggesting that it is distinct from movement-related broadband gamma synchronisation (reviewed in [55]). In the current study, sustained NBG oscillations (70–110 Hz) emerged in several cortico-basal ganglia structures in conjunction with dyskinetic behaviour induced by chronic L-DOPA treatment. Compared to other publications on the subject, rats were treated with a relatively low dose of L-DOPA (6 mg/kg s.c.) that is commonly used in behavioural studies [47–49]. In agreement with reports using a higher dose of L-DOPA (12–15 mg/kg) [15, 16, 18–20] NBG oscillations were detected in the motor cortex of the lesioned hemisphere in all rats as well as in the striatum and GPe, whereas the detection rate in the SNr was limited. In contrast, a recent study did not find an increase in NBG oscillations in 6-OHDA-lesioned rats after treatment with 7 mg/kg L-DOPA in a 3-week priming paradigm, despite clear dyskinetic behaviour [56]. The discrepancy with our data could depend on differences in the methods of spectral analysis, where the use of IRASA in the current study (see the “Time–Frequency Analysis of LFP Power” section) allowed the isolation of true oscillatory phenomena [42].

The induction of NBG oscillations upon activation of D1 or D2 receptors has so far only been reported in specific motor cortical areas of the frontal lobe and upon acute administration of dopamine receptor agonists [18, 19]. This is the first comprehensive investigation comparing the potency of chronically administered D1 and D2 receptor agonists at generating NBG oscillations in several cortical and subcortical areas. Moreover, whereas previous studies have used D2-class agonists with high activity on D3 receptors [19], we have here used the very selective D2 receptor agonist sumanirole. Stimulation of the D1 receptor elicited strong NBG oscillations not only in motor cortices but also in DMS, DLS, and GPe, that is, in all the recorded structures with the exception of SNr. Following treatment with the D2 agonist, NBG oscillations were elicited only in forelimb motor areas and DLS, but not in M1Tr, DMS, GPe, and SNr. In the latter structure, none of the tested treatments induced NBG oscillatory activity, indicating that the basal ganglia output via SNr is less prone to maintain these high-frequency oscillations. In the frequency domain, NBG oscillations were strongly modulated by the pharmacological treatments, where the mean frequency of the elicited NBG oscillations was highest after L-DOPA treatment and lowest after treatment with sumanirole. Similar modulations of broadband gamma oscillations have been reported in anaesthetised hemiparkinsonian rats, with higher peak frequencies after treatment with L-DOPA compared to the potent dopamine receptor agonist apomorphine [57].

In agreement with previous studies [18, 19], we found strong NBG oscillations in forelimb motor cortical areas, which were more prominent than in all other structures recorded and correlated strongly with dyskinesia severity (AIM scores). Interestingly, applying a D1 receptor antagonist on the cortical surface has been found to stop both LID and cortical NBG oscillations in hemiparkinsonian animals [18], strongly indicating a direct cortical involvement. Thus, Halje et al. [18] proposed that loss of cortical dopaminergic innervations may be a key predisposing factor for dyskinesia and that the resulting sensitivity to dopamine makes the cortical circuits prone to network resonance in the NBG frequency range. As a novel contribution, our data indicate a particularly prominent role of the D1 receptor in inducing these cortical NBG oscillations, which is in line with the critical role of D1 receptor supersensitivity in LID [26, 58]. Indeed, the D2 receptor agonist sumanirole was significantly less potent than SKF82958 at eliciting NBG oscillations. The superior potency of the D1 agonist might partly reflect the higher relative density of D1 receptors compared to D2 receptors in motor cortical regions [59], although further investigations are needed to clarify the specific cellular distribution of these two receptor classes in the cortex, and their changes following dopamine denervation. In the phase synchrony analysis, we found that NBG oscillations in the M1FL were leading motor cortical and striatal structures, with a stronger effect following treatment with the D1 receptor agonist (see further discussion below). Considering that M1FL showed the highest NBG detection rate, these findings point to this structure as a potential driver of these oscillations, at least for the circuits recorded in this study.

Most studies have so far focused on NBG oscillations in frontocortical motor areas, and it has remained unclear whether this rhythm could be induced in other cortical regions. For the first time, we here recorded NBG activity in a more caudal primary motor cortical region located in the parietal lobe (M1Tr), previously reported to be involved in trunk movements [60, 61]. In this area, the induction of NBG oscillations was considerably lower than in frontal motor cortical areas controlling forelimb movements, and this rhythm was not even detectable following D2 stimulation. It is interesting to consider that the strongest NBG oscillations were detected in motor regions related to forelimb movements, which are mainly engaged in fast hyperkinetic forms of dyskinetic behaviour in this animal model [36, 62]. After all treatments, NBG oscillations were markedly less pronounced in motor regions controlling trunk movements, which are engaged in slow twisting movements [36, 62]. Additionally, it is worth noting that treatment with the D2 receptor agonist did not induce NBG oscillations in the trunk motor area.

Another noteworthy difference between D1 and D2 receptor stimulation is that no NBG activity was detected in the GPe after D2 stimulation, in contrast to the high detection rate equally induced by the administration of the D1 receptor agonist or L-DOPA in this region. Treatment with L-DOPA has previously been reported to induce NBG oscillations in the GPe in 6-OHDA-lesioned rats [15, 16], but this is the first report comparing the effects of D1 and D2 receptor agonists on GPe oscillatory activities. We demonstrate that the effects of L-DOPA on pallidal NBG oscillations are mimicked by the D1 receptor agonist in terms of both detection rate and power, with no effects of the D2 receptor agonist, suggesting that these oscillations in GPe are clearly driven by D1 stimulation. It is interesting to note the almost identical patterns induced in all parameters of NBG activity in both GPe and M1Tr, especially considering that these regions had a tight functional coupling (discussed in further detail below). In consideration of the recently revealed detailed topography of cortico-basal ganglia-thalamic networks [63], it is worth noting that our electrode implants aimed at a rather caudal-lateral part of GPe (see Table 1). This might explain the tighter functional coupling found between GPe and the more caudally located trunk motor area compared to rostral forelimb areas.

### Effects of D1 and D2 Receptor Agonists on Theta Oscillatory Activity

In conjunction with motor state transitions occurring after the three pharmacological treatments, a prominent increase of theta oscillations (5–10 Hz) was detected in several basal ganglia structures of both the intact and lesioned hemisphere. In the deeper nuclei of the basal ganglia (GPe and SNr), theta oscillations were strongly increased by D2 stimulation, whereas the effects of D1 stimulation and L-DOPA on this rhythm were less pronounced. Alterations in the spectral contents of slow oscillatory activities have previously been reported in deep basal ganglia nuclei in PD patients after dopamine replacement therapy [12, 13]. Recent studies in animal models of LID have confirmed the presence of theta oscillations in the basal ganglia after treatment with L-DOPA [15–17]. In accordance with our findings, these studies show a prominent increase of theta oscillations in both hemispheres, particularly present during periods of dyskinesia. However, it was proposed that some of the spectral changes are most likely associated with the more active behavioural state rather than to dyskinetic motor signs per se [15, 16]. Nevertheless, recording from the subthalamic nucleus in PD patients, Alonso-Frech and colleagues [12] have shown a strong relationship between theta oscillations and dyskinesia. For example, when dyskinesias were asymmetrically expressed, theta oscillations were only present in the contralateral hemisphere. Moreover, in patients with diphasic dyskinesia, theta oscillations occurred in strict temporal coincidence with the involuntary movements. In line with these data, our results show significant increases in theta oscillations during the expression of dyskinesia, the strongest effects being seen in GPe and SNr of the lesioned hemisphere after treatment with sumanirole. These data suggest an important role of D2 receptor stimulation in the induction of theta oscillations.

It remains to be established what particular form of PD dyskinesias is mimicked by D2 receptor agonists inducing pallidal theta oscillations. Whereas all current animal models of LID (as treated with L-DOPA) reproduce choreiform “on” dyskinesias [28], PD patients exhibit a larger variety of dyskinetic manifestations that may depend on a differential engagement of D1 vs. D2 receptors. Accordingly, it has been suggested that “on” and biphasic dyskinesias are differentially dependent on D1 vs. D2 receptors, respectively [64]. Taking into consideration that increases in theta activity have been reported in PD patients experiencing diphasic dyskinesias [12], it is tempting to speculate that the D2 agonist-induced AIMs in our animal model mimic diphasic dyskinesia rather than the “on” pattern. Another aspect to consider is the relative representation of fast hyperkinetic vs. slow dystonic-like components in the involuntary movements. Indeed, our recent studies in L-DOPA-naïve 6-OHDA-lesioned mice show that, in the absence of D1 stimulation, D2 receptors selectively mediate dystonic forms of dyskinesia [62]. Interestingly, an association between dystonic motor manifestations and theta oscillations has been found in primary dystonia patients using pallidal recordings (reviewed in [9]). In our animal model, sumanirole-induced dyskinetic behaviours were characterised by a predominant representation of slow components (axial AIMs) relative to fast hyperkinetic components (limb and orolingual AIMs). Moreover, the analysis of treatment-induced rotational behaviour revealed slower turning speed after treatment with sumanirole compared with SKF82958. Taken together, our data suggests that the stronger increase in pallidal and nigral theta oscillations induced by sumanirole may underlie the different pattern of dyskinetic behaviours induced by D2 compared to D1 receptor agonists.

### Phase Analysis of NBG Oscillations

To study the network dynamics of NBG oscillations, we examined the changes in inter-structure connectivity during peak dyskinesia that, in parallel to intrinsic network changes induced by the dopaminergic denervation and the pharmacotherapy, may facilitate the transmission of these oscillations between different brain regions [54, 65, 66]. Phase analysis revealed that NBG oscillations in the M1FL were leading motor cortical and striatal structures, with a stronger effect following treatment with the D1 receptor agonist, which suggests that this region might be a key driver of these oscillations. It is, however, important to keep in mind that both cortex and the basal ganglia are under strong influence of thalamic input, which potentially could be a common pacemaker of these high-frequency oscillations in several parts of the circuit (reviewed in [23]). In line with the consistent phase differences, primary motor cortical areas (M1FL and M1Tr) showed strong functional connectivity intracortically as well as to the striatum upon D1 receptor stimulation, independent of the effects on oscillatory power. Interestingly, M1Tr and RFA also showed functional connectivity to GPe, supporting the theory that high-frequency oscillations are intrinsically generated in the cortical network, from which they spread to subcortical structures (see [23]), with a more pronounced role of the D1 receptor (as covered above). Collectively, these findings suggest a fundamental role of altered D1 receptor modulation of cortico-basal ganglia connectivity in the emergence of NBG oscillations underlying LID.

## Concluding Remarks

**Fig. 8:**
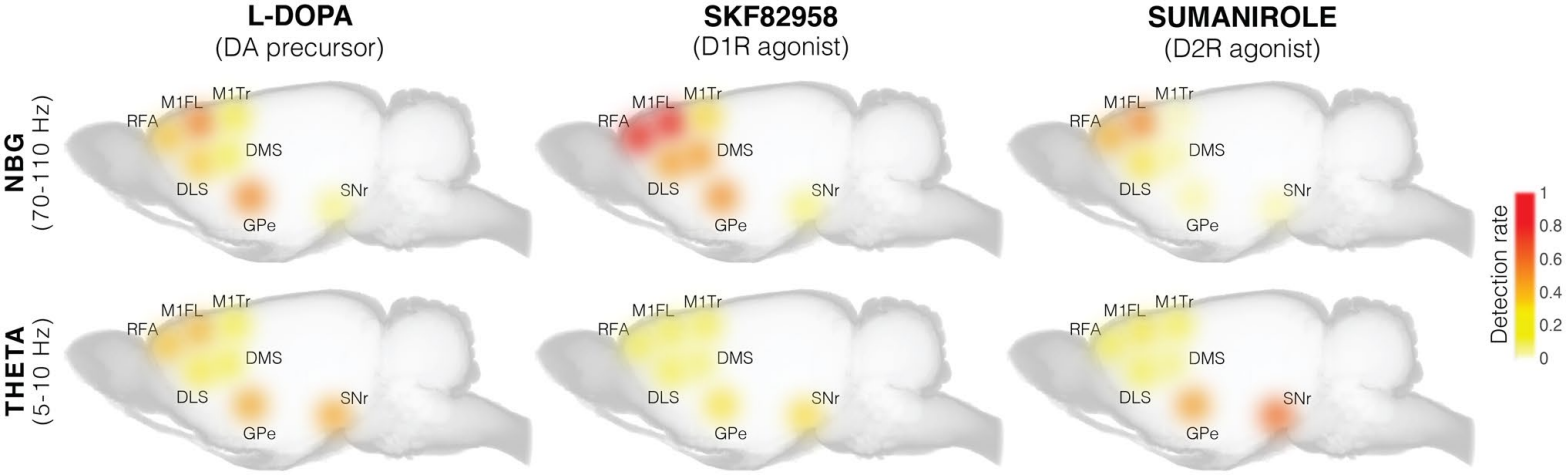
Graphical summary representing treatment effects on cortico-basal ganglia oscillations during peak dyskinesia. Effects of L-DOPA, the D1R agonist SKF82958, or the D2R agonist sumanirole on NBG (top row) or theta oscillations (bottom row) recorded in the lesioned hemisphere during peak dyskinesia severity. The detection rate within each structure is represented using the indicated colour scale. Abbreviations: rostral forelimb area (RFA), primary motor cortex forelimb area (M1FL), primary motor cortex trunk area (M1Tr), dorsomedial striatum (DMS), dorsolateral striatum (DLS), globus pallidus pars externa (GPe), substantia nigra pars reticulata (SNr), and narrowband gamma (NBG)

The present study represents the first comprehensive investigation of LFP oscillatory patterns in the cortico-basal ganglia network induced by D1 vs. D2 receptor agonists in a well-established animal model of LID. Our data reveal a strong effect of D1 receptor stimulation in inducing network-wide NBG oscillations, as SKF82958 was even more potent than L-DOPA in several of the recorded structures (particularly in frontocortical motor regions controlling forelimb movements). The effect of D2 receptor stimulation was instead characterised by weaker and topographically more restricted NBG activity, while strong theta oscillations were induced in the GPe and SNr. A graphic summary of these main results is provided in Fig. 8. Among all the recorded structures, the GPe was the one exhibiting the largest dissociation between the effects of D1 vs D2 receptor stimulation, as the latter induced no NBG but a relatively strong theta rhythm. These results suggest that LFP spectral contents in pallidal regions may be used to parse the relative contribution of D1 vs. D2 receptors in mediating different forms of dyskinesias in PD. Because D1 vs. D2 oscillatory patterns segregated with partially different dyskinetic behaviours, our results moreover encourage further investigations on the relationship between specific dyskinetic manifestations, their oscillatory fingerprints, and the underlying circuit dysfunctions in PD.

## Supplementary Information

Below is the link to the electronic supplementary material.

Supplementary file1 (DOCX 12.1 MB)

Supplementary file2 (XLSX 41.9 KB)

Supplementary file3 (PDF 533 KB)

Supplementary file4 (PDF 542 KB)

Supplementary file5 (PDF 559 KB)

Supplementary file6 (PDF 585 KB)

Supplementary file7 (PDF 594 KB)
