# Supplementary material for: Distinctive Effects of D1 and D2 Receptor Agonists on Cortico-Basal Ganglia Oscillations in a Rodent Model of L-DOPA-Induced Dyskinesia
Source: Neurotherapeutics. 2022 Nov 7;20(1):304–24. doi: 10.1007/s13311-022-01309-5 (PMC10119363; doi:10.1007/s13311-022-01309-5)
Supplement: Supplementary file 1 — Supplementary file1 (DOCX 12.1 MB) [file 13311_2022_1309_MOESM1_ESM.docx]

**Supplemental Methods**

*Tyrosine hydroxylase (TH) staining*

To verify the extent of dopaminergic degeneration, brain sections through the midbrain of 6 animals were immunostained for tyrosine hydroxylase (TH; a dopamine cell marker) (Fig. 1B). Sections were rinsed in 0.02 M phosphate buffered saline containing potassium (KPBS), quenched in 3% hydrogen peroxide (H_2_O_2_) diluted in 10% methanol in KPBS (15 min) and rinsed in KBPS triton x-100 0.1 % (KPBS-T). Then, sections were pre-incubated with 5% normal goat serum (NGS) in KPBS-T for 1 h followed by incubation with primary antibody rabbit anti-TH (1:1000 in 5 % NGS/KPBS-T; P40101-150, Pel-Freez Biologicals, Rogers, AR, USA) overnight at 4°C. On the following day, sections were rinsed in KPBS-T and 5 % NGS/KPBS-T before incubating with biotinylated goat anti-rabbit antibody (1:200 in 5 % NGS/KPBS-T, BA 1000; Vector Laboratories, Burlingame, CA, USA) for 1 h at room temperature. Thereafter, all sections were rinsed in KPBS-T, incubated in Avidin/Biotin Elite (ABC Kit; Vectastain, Vector Laboratories, CA, USA) for 1 h, rinsed in KPBS-T and stained with 3,3'-Diaminobenzidine (DAB) and 3% H_2_O_2_. Then, sections were rinsed with KPBS and mounted on chromalum-gelatin coated slides (Thermo Scientific, Menzel-Gläser, Braunschweig, Germany). Lastly, mounted sections were dehydrated in ascending ethanol concentrations followed by xylene, and coverslipped with DPX mounting medium (Sigma-Aldrich). The immunostained sections were inspected under the microscope. All animals exhibited a nearly complete loss of dopaminergic neurons in the substantia nigra pars compacta ipsilateral to the toxin injections, as expected from this MFB lesion model (Andersson et al., 1999).

*Dyskinesia dose-response curves*

A pilot experiment was carried out in a separate group of animals to decide on the dose of each treatment (Supplemental Fig. S1). 30 adult female Sprague-Dawley rats underwent unilateral nigrostriatal dopamine lesions and three weeks after the lesion, animals with severe (>85%) unilateral dopamine denervation were selected using the cylinder test of forelimb use asymmetry (see section 2.4 for details). The animals were primed with 6 mg/kg L-DOPA and 12 mg/kg benserazide as described in section 2.4 and divided into two groups. The first group of animals (n = 12) was administered with SKF82958 every second day for one week in a dose of 1 (n = 4), 0.5 (n = 8), and 0.05 mg/kg (n = 11), respectively. The second group of animals (n = 18) was administered with sumanirole every second day for one week in a dose of 4, 3, and 2 mg/kg, respectively (see *section* 2.3 for drug details). During dyskinesia testing sessions, animals were placed individually in a transparent plastic cage (42.5 x 26.5 cm) and allowed to habituate for 15 min. Then, animals were injected with either SKF82958 or sumanirole according to their treatment group and AIMs were scored according to section 2.5 for monitoring periods of 1 min every 20 min for a total of 160 min (group 1) or 180 min (group 2) following drug injection.

**Supplemental Fig. S1** **Schematic representation of the study design of dyskinesia dose-response experiments.** Rats sustained 6-OHDA-lesions in the right MFB and were primed with L-DOPA before being divided in two groups to receive treatment with either SKF82958 (1, 0.5, and 0.05 mg/kg s.c.) or sumanirole (SUM; 4, 3, and 2 mg/kg s.c.). Rats were left drug free for one day before moving from the highest to the lowest drug dose tested. Dyskinesia ratings were carried out once for each drug dose tested

**Supplemental Fig. S2** **Dose-response curves of the** **D1R agonist, SKF82958 and the D2R agonist, sumanirole on inducing abnormal involuntary movements (AIMs) in non-implanted 6-OHDA lesioned animals. A.** SKF82958 was tested in doses of 0.05 (n = 11), 0.5 (n = 8) and 1 mg/kg s.c. (n = 4). The global AIMs scores are presented per monitoring period as a function of time. (Mixed-effects model: F(treatment) =44; F(time)_7,77_ = 13, p < 0.001; F(interaction)_14,46_ = 29, p < 0.001; Tukey’s post hoc: *p<0.05 vs. 0.5 mg/kg, ^p<0.05 vs. 1 mg/kg). **B.** SUM was tested in doses of 2, 3, and 4 mg/kg s.c. (n = 18). The global AIMs scores are presented per monitoring period as a function of time. (Mixed-effects model: F(treatment)_2,34_ =4, p = 0.027; F(time)_4,68_ = 12, p < 0.001; F(interaction)_7,106_ = 3, p = 0.002; Tukey’s post hoc: *p<0.05 vs. 3 mg/kg, ^p<0.05 vs. 4 mg/kg)

From the dose-response curves, it is shown how higher doses of SKF82958 could induce dyskinesia over longer time periods in non-implanted rats, comparable to the time course induced by L-DOPA or sumanirole, whereas higher doses of sumanirole did not induce more severe dyskinesia, but rather prolonged the time course even further (Supplemental Fig. S2). This suggests a ceiling to the effects of sumanirole on inducing dyskinesia.

**Supplemental Table S1** Typical limit values for fitted parameters of parametric model and goodness of fit (R2).

| Band | R2 | A (peak hight) low/high | B (peak frequency) low/high | C (peak width) low/high |
| --- | --- | --- | --- | --- |
| Beta | 0.2 | 2/NA | 11/NA | 1/10 |
| NBG | 0.3 | 2/NA | 69/119 | 1/20 |
| Theta | 0.35 | 3.5/NA | 4/NA | 0.1/2 |

*Abbreviations: narrowband gamma (NBG)*

**Supplemental Fig. S3 Detection of oscillatory activity (A)** Examples of LFP power spectra (grey) from 8 sec of data are shown for L-DOPA (left), SKF82958 (middle), or SUM (right). Green or red lines show the corresponding fitted function $y\left( f \right)=Ae^{-\left( \frac{f-B}{C} \right)^{2}}+Df+E$. Functions with parameter values and goodness-of-fit within given limits were counted as successful peak detections and marked in green. **(B)** Spectrograms (same as in Fig. 4B from M1FL). Each green point marks an 8-sec bin with successful peak detection. *Abbreviations: Primary motor cortex forelimb area (M1FL)*

**Results**

**Supplemental Fig. S4** **Effects of the D1R agonist, SKF82958 and the D2R agonist, sumanirole on inducing abnormal involuntary movements (AIMs) and open-field motions in comparison to L-DOPA.** **(A-C)** Total scores of AIM subtypes calculated as the sum of total drug effect (0-180 min post injection) upon treatment with L-DOPA (blue), SKF82958 (green), and SUM (red). **(A)** Axial total AIM scores (Mixed-effects model: F(treatment)1.365,15.70 = 7.43, p = 0.01). **(B)** Limb total AIM scores (Mixed-effects model: F(treatment)1.584,18.21 = 9.39, p = 0.003). **(C)** Orolingual total AIM scores (Mixed-effects model: F(treatment)1.422,16.36 = 22.72, p < 0.001). **(D)** Axial, limb, and orolingual AIM scores are here represented as a percentage of the total ALO AIM scores after each treatment (Mixed-effects model: F(treatment)_2,12_ = 0.2, p = 0.757; F(AIM subtype)_2,16_ = 146, p < 0.001; F(interaction)_2,14_ = 23, p < 0.001). **(E-G)** Peak scores of AIM subtypes calculated as sum during peak dyskinesia upon treatment with L-DOPA (40-80 min), SKF82958 (20-60 min), and SUM (40-80 min). **(E)** Axial peak AIM scores (Mixed-effects model: F(treatment)_1.167,8.756_ = 58.21, p < 0.0001). **(F)** Limb peak AIM scores (Mixed-effects model: F(treatment)_1.284,9.629_ = 20.24, p = 0.0008). **(G)** Orolingual peak AIM scores (Mixed-effects model: F(treatment)_1.545,17.77_ = 23.56, p < 0.0001). **(H)** Axial, limb, and orolingual AIM scores are here represented as a percentage of the peak ALO AIM scores after each treatment (Mixed-effects model: F(treatment)_2,12_ = 0.03, p = 0.946; F(AIM subtype)_2,16_ = 123, p < 0.001; F(interaction)_3,20_ = 12, p < 0.001). **(I-L)** Distinctive motion patterns are observed in the open field arena in 6-OHDA lesioned rats receiving different treatments. **(I)** Time course of fraction of total area visited per min upon treatment with veh (grey), L-DOPA (blue), SKF82958 (green), and SUM (red). Coloured bars mark peak period chosen for further analysis. **(J)** Total fraction of total area visited calculated from 0-180 min post injection (Mixed-effects model: F(treatment)_1.668,12.23_ = 37.27, p < 0.001). **(K)** Peak fraction of total area visited calculated during periods of peak dyskinesia (cf. shaded area in I) (Mixed-effects model: F(treatment)_2.166,22.38_ = 36.28, p < 0.001). **(L)** Traces representing the animal movement pattern inside the circular arena within 2 min recording at the peak of drug effect (SKF82958: 40-42 min; L-DOPA, SUM, veh: 60-62 min). Tukey’s post hoc: &p<0.05 vs. veh, *p<0.05 vs. L-DOPA, #p<0.05 vs. SKF82958. Treatments: vehicle (veh; grey, n = 9), L-DOPA (blue, 6.0 mg/kg s.c., n = 9), SKF82958 (green, 0.05 mg/kg s.c., n = 9), sumanirole (SUM; red, 2.0 mg/kg s.c., n = 8)

Examining the individual AIM subtypes revealed that all axial, limb, and orolingual (ALO) subtypes were lower after treatment with sumanirole compared to both L-DOPA and SKF82958 (Supplemental Fig. S4E-G; Axial: -36 % vs. L-DOPA, *p = 0.003; -47 % vs. SKF82958, #p < 0.001; Limb: -44 % vs. L-DOPA, *p = 0.001; -49 % vs. SKF82958, #p = 0.005; Orolingual: -81 % vs. L-DOPA, *p = 0.005; -70 % vs. SKF82958, #p < 0.001), whereas dyskinesias induced by SKF82958 showed higher axial peak AIM scores compared to L-DOPA (Supplemental Fig. S4E; +20 % vs. L-DOPA, *p = 0.014). When considering the percentage of total AIMs, orolingual AIMs were lower in sumanirole treated animals compared to L-DOPA (Supplemental Fig. S4H; *p = 0.006 vs. L-DOPA), whereas axial AIMs were higher after treatment with SKF82958 (Supplemental Fig. S4H; *p = 0.027 vs. L-DOPA). In addition, the overall motions in the test arena induced by the treatments were higher for both dopamine agonists compared to L-DOPA, but also higher for sumanirole compared to SKF82958 when taking the total time into consideration (Supplemental Fig. S4I-L; Total: *p = 0.049 SKF82958 vs. L-DOPA, *p = 0.003 SUM vs. L-DOPA, #p = 0.03 SUM vs. SKF82958; Peak: *p = 0.006 SUM vs. L-DOPA).

**Supplemental Fig. S5 Temporal evolution of treatments effects on NBG and theta LFP oscillations.** Detection rates of NBG **(A)** and theta **(B)** oscillations in the M1FL and GPe, respectively, of both the intact (grey shaded, left panel) and lesioned (right panel) hemisphere during representative timepoints of the dyskinetic behaviour upon treatment with veh (grey), L-DOPA (blue), SKF82958 (green), and SUM (red). Dots mark the mean detection rate per animal. Abbreviations: primary motor cortex forelimb area (M1FL), globus pallidus pars externa (GPe)

**Supplemental Fig. S6** Effects of treatments on NBG oscillations in the intact hemisphere during peak dyskinesia. Within each structure of the intact hemisphere, the effects of veh (grey), L-DOPA (blue), SKF82958 (green), and SUM (red) on the detection rate of NBG oscillations were compared during peak dyskinesia. Dots mark the mean per animal. *Abbreviations: Rostral forelimb area (RFA), primary motor cortex forelimb area (M1FL), primary motor cortex trunk area (M1Tr), dorsomedial striatum (DMS), dorsolateral striatum (DLS), globus pallidus pars externa (GPe), and substantia nigra pars reticulata (SNr)*

**

**Supplemental Fig. S7** **Effects of the treatments on cortico-basal ganglia NBG oscillations in the lesioned hemisphere at time points with comparable levels of dyskinesia.** Within each structure of the lesioned hemisphere NBG oscillations hemisphere were investigated at time points with comparable global AIM scores between treatments, that is L-DOPA (blue; 130-140 min), SKF82958 (green; 70-80 min), SUM (red; 70-80 min), and vehicle (grey; 70-80 min). **(A)** Time course of global AIM scores as presented in Fig. 3A. Coloured bars mark time periods of comparable levels of dyskinesia chosen for further analysis. **(B)** Detection rate of NBG oscillations (Mixed-effects model: χ²(treatment)_2_ = 10.476, p = 0.005; χ²(structure)_6_ = 4399.7, p < 0.001; χ²(interaction)_12_ = 1934, p < 0.001). **(C)** Absolute power (dB) of the detected peaks (Mixed-effects model: F(treatment)_2,47.9_ = 2.377, p = 0.104; F(structure)_5,7877_ = 166.2, p < 0.001; F(interaction)_5,7871.8_ = 164.9, p < 0.001). **(D)** Frequency (Hz) of the detected peaks (Mixed-effects model: F(treatment)_2,34.3_ = 23.35, p < 0.001; F(structure)_5,7846.2_ = 83.45, p < 0.001; F(interaction)_5,7856_ = 10.46, p < 0.001). Dots mark the mean per animal. Scheffe’s post hoc: &p<0.05 vs. veh, *p<0.05 vs. L-DOPA, #p<0.05 vs. SKF82958, ^p<0.05 vs. SUM. Abbreviations: Rostral forelimb area (RFA), primary motor cortex forelimb area (M1FL), primary motor cortex trunk area (M1Tr), dorsomedial striatum (DMS), dorsolateral striatum (DLS), globus pallidus pars externa (GPe), and substantia nigra pars reticulata (SNr)

Oscillatory NBG activity is here investigated at time points with comparable dyskinesia severity between treatments (Supplemental Fig. S7A). Compared to the effects seen during peak dyskinesia (see Fig. 5), the NBG detection rate induced by L-DOPA and SKF82959 was generally lower (Supplemental Fig. S7B). In M1FL and RFA, the detection rate of NBG oscillations was increased significantly by SKF82958 and sumanirole compared to L-DOPA (Supplemental Fig. S7B; RFA: OR_L-DOPA/SKF82958_ = 0.07±0.04, *p < 0.001; OR_L-DOPA/SUM_ = 0.06±0.04, *p < 0.001; M1FL: OR_L-DOPA/SKF82958_ = 0.24±0.14, *p < 0.001), although the difference between the two dopamine agonists did not reach significance in these cortical structures. In line with the effects seen during peak dyskinesia severity (see Fig. 5A), SKF82958 induced higher detection rates compared to sumanirole in the M1Tr, DMS, and GPe (Supplemental Fig. S7B; M1Tr: OR_SKF82958/SUM_ = 56.83±34.68, *p < 0.001; DMS: OR_SKF82958/SUM_ = 64.41±38.79, *p < 0.001; GPe: OR_SKF82958/SUM_ = 30.9±19.12, *p = 0.002). In GPe, the detection rate was significantly higher for both SKF82958 and L-DOPA compared to sumanirole (GPe: OR_L-DOPA/SUM_ = 47.95±30.97, *p < 0.001), as seen during peak AIM severity (see Fig. 5A). Moreover, similarly to what was observed during peak dyskinesia, there was no difference between treatments in NBG detection rates in DLS and SNr (Supplemental Fig. S7B). In fact, in the SNr, the detection rate of NBG oscillations was below the threshold for all treatments (Supplemental Fig. S7B). When examining the absolute power of the detected peaks, the highest values were generally measured upon treatment with SKF82958, reaching significance compared to both L-DOPA and sumanirole in M1FL (Supplemental Fig. S7C; ratio_L-DOPA/SKF82958_ = 0.71±0.05, *p < 0.001; ratio_SKF82958/SUM_ = 1.63±0.11, *p < 0.001). As to the NBG oscillation frequencies (Supplemental Fig. S7D), the pattern of treatment differences was similar to that observed at peak dyskinesia severity, with generally higher values for L-DOPA and SKF82958 compared with sumanirole, reaching statistical significance in all structures where NBG oscillations were detected (Supplemental Fig. S7D; see ^p < 0.001).

**Supplemental Fig. S8** **Correlation of AIM scores and LFP oscillations.** **(A-C)** Within each structure of the lesioned hemisphere, global AIM scores were correlated with the total power in the NBG **(A)** or theta **(B)** frequency bands across treatments. Individual data points of the correlations (Spearman’s) represent the 1 min monitoring periods of AIM ratings in the individual recordings (being 1 min every 5 min post injection for the first 20 min and then every 10 min for the rest of the testing session, which lasted 180 min). The NBG power (dB) represents the average value during the 1-min monitoring period. Data points are color-coded according to treatment (veh (grey), L-DOPA (blue), SKF82958 (green), and SUM (red)) and shades of grey represent the density of data points. Abbreviations: Spearman’s correlation coefficient (Rho), Rostral forelimb area (RFA), primary motor cortex forelimb area (M1FL), primary motor cortex trunk area (M1Tr), dorsomedial striatum (DMS), dorsolateral striatum (DLS), globus pallidus pars externa (GPe), and substantia nigra pars reticulata (SNr)

**Supplemental Table S2** Correlations between NBG power and AIMs subtypes.

| **Structure** | **Axial** | | **Limb** | | **Orolingual** | |
| --- | --- | --- | --- | --- | --- | --- |
|  | Rho | p | Rho | p | Rho | p |
| RFA | 0.659 | < 0.001 | 0.635 | < 0.001 | 0.553 | < 0.001 |
| M1FL | 0.687 | < 0.001 | 0.703 | < 0.001 | 0.579 | < 0.001 |
| M1Tr | 0.426 | < 0.001 | 0.434 | < 0.001 | 0.406 | < 0.001 |
| DMS | 0.538 | < 0.001 | 0.545 | < 0.001 | 0.470 | < 0.001 |
| DLS | 0.507 | < 0.001 | 0.517 | < 0.001 | 0.437 | < 0.001 |
| GPe | 0.625 | < 0.001 | 0.640 | < 0.001 | 0.607 | < 0.001 |
| SNr | 0.324 | < 0.001 | 0.341 | < 0.001 | 0.271 | < 0.001 |

Correlations were carried out as described in Supplemental Fig. S8, although now using the AIM subscores (axial, limb, or orolingual) instead of the global AIM scores per monitoring period. Values highlighted in blue mark significant correlations (p < 0.05). *Abbreviations: Spearman’s correlation coefficient (Rho), Rostral forelimb area (RFA), primary motor cortex forelimb area (M1FL), primary motor cortex trunk area (M1Tr), dorsomedial striatum (DMS), dorsolateral striatum (DLS), globus pallidus pars externa (GPe), and substantia nigra pars reticulata (SNr)*

**Supplemental Table S3** Correlations between theta power and AIMs subtypes.

| **Structure** | **Axial** | | **Limb** | | **Orolingual** | |
| --- | --- | --- | --- | --- | --- | --- |
|  | Rho | p | Rho | p | Rho | p |
| RFA | 0.063 | 0.059 | 0.118 | < 0.001 | 0.127 | < 0.001 |
| M1FL | 0.182 | < 0.001 | 0.185 | < 0.001 | 0.231 | < 0.001 |
| M1Tr | -0.002 | 0.946 | -0.041 | 0.232 | 0.061 | 0.073 |
| DMS | 0.050 | 0.108 | 0.055 | 0.077 | 0.133 | < 0.001 |
| DLS | 0.054 | 0.048 | 0.077 | 0.005 | 0.109 | < 0.001 |
| GPe | 0.316 | < 0.001 | 0.306 | < 0.001 | 0.351 | < 0.001 |
| SNr | -0.014 | 0.718 | -0.058 | 0.136 | -0.053 | 0.179 |

Correlations were carried out as described in Supplemental Fig. S8, although now using the AIM subscores (axial, limb, or orolingual) instead of the global AIM scores per monitoring period. Values highlighted in blue mark significant correlations (p < 0.05). *Abbreviations: Spearman’s correlation coefficient (Rho), Rostral forelimb area (RFA), primary motor cortex forelimb area (M1FL), primary motor cortex trunk area (M1Tr), dorsomedial striatum (DMS), dorsolateral striatum (DLS), globus pallidus pars externa (GPe), and substantia nigra pars reticulata (SNr)*

**Supplemental Fig. S9** **Correlation between open field motions and theta oscillations.** **(A-B)** Overall motions in the open field (represented by the fraction of total area visited) were correlated with the LFP power in the theta frequency band in the GPe **(A)** and SNr **(B)** of both the intact (top row) and the lesioned hemisphere (bottom row) across treatments (first column) and following each of the treatments L-DOPA (blue), SKF82958 (green), and SUM (red). Individual data points of the correlations (Spearman’s) correspond to the same 1-min monitoring periods considered for the AIM ratings (see Supplemental Fig. S8). Shades of grey represent the density of data points. Abbreviations: Spearman’s correlation coefficient (Rho), Globus pallidus pars externa (GPe), substantia nigra pars reticulata (SNr)


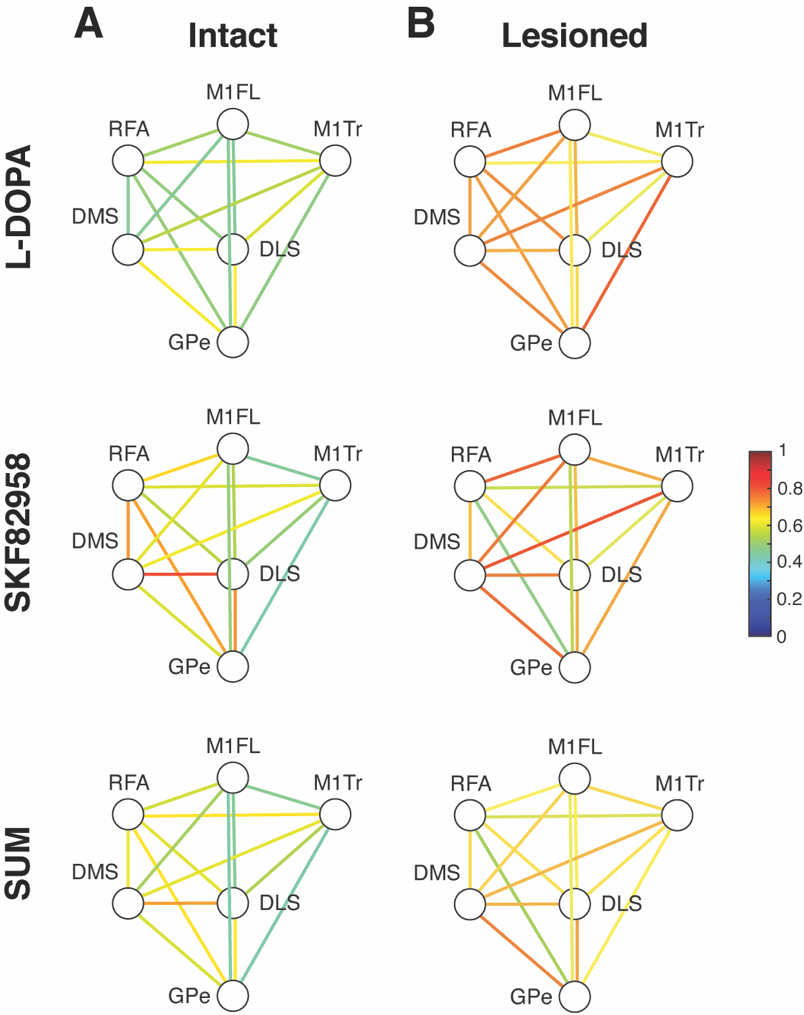


**Supplemental Fig. S10** **Functional connectivity in the NBG frequency band.** Diagrams of NBG functional connectivity for L-DOPA (top panel), SKF82958 (middle panel), and sumanirole (bottom panel), as represented by the magnitude of the resultant vector of the phase distributions in the intact **(A)** and lesioned **(B)** hemisphere (see colour scale). In the shown diagrams, all comparisons of functional connectivity between treatments are significant for each structure pair (Wilcoxon signed-rank test: z = -97.4, p < 0.001; see Supplemental Table S6). Abbreviations: Rostral forelimb area (RFA), primary motor cortex forelimb area (M1FL), primary motor cortex trunk area (M1Tr), dorsomedial striatum (DMS), dorsolateral striatum (DLS), and globus pallidus pars externa (GPe)­­­

**Supplementary Table S4** Selected detailed statistical information from multiple comparison post hoc tests for detection rate. Find the complete statistical information in Supplemental statistical materials.

| Detection rate | | | | | | | | | |
| --- | --- | --- | --- | --- | --- | --- | --- | --- | --- |
| Frequency range | **Hemisphere** | **Structure** | **Contrast** | **Odds ratio** | **SE** | **LCL** | **UCL** | **z-ratio** | **p-value** |
| Theta | Lesioned | RFA | veh / L-DOPA | 0.226 | 0.0364 | 0.0902 | 0.568 | -10.305 | <.0001 |
| Theta | Lesioned | RFA | L-DOPA / SKF82958 | 3.883 | 0.5767 | 1.6613 | 9.075 | 7.969 | <.0001 |
| Theta | Lesioned | RFA | L-DOPA/SUM | 3.957 | 0.6252 | 1.6037 | 9.762 | 7.61 | <.0001 |
| Theta | Lesioned | DMS | veh / L-DOPA | 0.323 | 0.0527 | 0.1271 | 0.821 | -7.983 | <.0001 |
| Theta | Intact | RFA | veh / L-DOPA | 0.184 | 0.0338 | 0.0644 | 0.525 | -10.465 | <.0001 |
| Theta | Intact | RFA | L-DOPA / SUM | 4.999 | 0.8693 | 1.8499 | 13.507 | 7.938 | <.0001 |
| Theta | Intact | M1FL | veh / L-DOPA | 0.344 | 0.0592 | 0.1289 | 0.92 | -7.531 | <.0001 |
| Theta | Intact | M1FL | L-DOPA / SKF82958 | 3.328 | 0.5377 | 1.3216 | 8.38 | 6.027 | 0.0201 |
| Theta | Intact | M1FL | L-DOPA / SUM | 4.585 | 0.7761 | 1.7423 | 12.064 | 7.645 | <.0001 |
| Theta | Intact | DLS | veh / L-DOPA | 0.337 | 0.0539 | 0.1349 | 0.84 | -8.235 | <.0001 |
| Theta | Intact | DLS | L-DOPA / SKF82958 | 3.821 | 0.6148 | 1.5231 | 9.585 | 6.909 | 0.0007 |
| Theta | Intact | DLS | L-DOPA / SUM | 7.991 | 1.3614 | 3.0181 | 21.16 | 10.858 | <.0001 |

Abbreviations: Standard error (SE), Lower control limit (LCL), Upper control limit (UCL), Rostral forelimb area (RFA), primary motor cortex forelimb area (M1FL), dorsomedial striatum (DMS), and dorsolateral striatum (DLS)

**Supplementary Table S5** Selected detailed statistical information from multiple comparison post hoc tests for peak frequency. Find the complete statistical information in Supplemental statistical materials.

| **Frequency** | | | | | | | | | |
| --- | --- | --- | --- | --- | --- | --- | --- | --- | --- |
| **Frequency range** | **Hemisphere** | **Structure** | **Contrast** | **Estimate/ratio** | **SE** | **LCL** | **UCL** | **z-ratio** | **p-value** |
| NBG | Lesioned | DLS | L-DOPA / SKF82958 | 5.91 | 0.814 | 1.94 | 9.87 | 7.255 | <.0001 |
| NBG | Lesioned | DLS | L-DOPA / SUM | 11.14 | 0.89 | 6.81 | 15.47 | 12.517 | <.0001 |
| NBG | Lesioned | DLS | SKF82958 / SUM | 5.23 | 0.834 | 1.18 | 9.29 | 6.276 | 0.0003 |
| NBG | Lesioned | DMS | L-DOPA / SKF82958 | 7.5 | 0.817 | 3.53 | 11.48 | 9.187 | <.0001 |
| NBG | Lesioned | GPe | L-DOPA / SKF82958 | 7.33 | 0.816 | 3.36 | 11.3 | 8.983 | <.0001 |
| NBG | Lesioned | M1FL | L-DOPA / SKF82958 | 7.01 | 0.812 | 3.05 | 10.96 | 8.626 | <.0001 |
| NBG | Lesioned | M1FL | L-DOPA / SUM | 12.81 | 0.887 | 8.49 | 17.13 | 14.436 | <.0001 |
| NBG | Lesioned | M1FL | SKF82958 / SUM | 5.8 | 0.831 | 1.76 | 9.85 | 6.979 | <.0001 |
| NBG | Lesioned | M1Tr | L-DOPA / SKF82958 | 7.73 | 0.817 | 3.75 | 11.71 | 9.461 | <.0001 |
| NBG | Lesioned | RFA | L-DOPA / SKF82958 | 6.29 | 0.814 | 2.32 | 10.25 | 7.72 | <.0001 |
| NBG | Lesioned | RFA | L-DOPA / SUM | 11.84 | 0.891 | 7.51 | 16.18 | 13.297 | <.0001 |
| NBG | Lesioned | RFA | SKF82958 -/SUM | 5.55 | 0.834 | 1.5 | 9.61 | 6.663 | 0.0001 |

Abbreviations: Standard error (SE), Lower control limit (LCL), Upper control limit (UCL), Rostral forelimb area (RFA), primary motor cortex forelimb area (M1FL), primary motor cortex trunk area (M1Tr), dorsomedial striatum (DMS), dorsolateral striatum (DLS), and globus pallidus pars externa (GPe)

**Supplementary Table S6** Selected detailed statistical information from multiple comparison post hoc tests for functional connectivity. Find the complete statistical information in Supplemental statistical materials.

| **Functional connectivity** | | | | | |
| --- | --- | --- | --- | --- | --- |
| **Frequency range** | **Hemisphere** | **Structure**  **pair** | **Contrast** | **%Change** | **p-value** |
| NBG | Lesioned | M1FL - M1Tr | SKF82958 / SUM | 11.5226925 | <0.0001 |
| NBG | Lesioned | RFA - M1FL | SKF82958 / SUM | 27.6521628 | <0.0001 |
| NBG | Lesioned | RFA - M1Tr | SKF82958 / SUM | 2.03526828 | <0.0001 |
| NBG | Lesioned | M1FL - DLS | SKF82958 / SUM | 10.8243068 | <0.0001 |
| NBG | Lesioned | M1FL - DMS | SKF82958 / SUM | 12.9930214 | <0.0001 |
| NBG | Lesioned | M1Tr - DLS | SKF82958 / SUM | 5.44495267 | <0.0001 |
| NBG | Lesioned | M1Tr - DMS | SKF82958 / SUM | 16.266178 | <0.0001 |
| NBG | Lesioned | RFA - DLS | SKF82958 / SUM | 14.7592883 | <0.0001 |
| NBG | Lesioned | RFA - DMS | SKF82958 / SUM | 16.4541079 | <0.0001 |
| NBG | Lesioned | DLS - GPe | SKF82958 / SUM | 6.23826601 | <0.0001 |
| NBG | Lesioned | DLS - GPe | SUM / L-DOPA | 6.91882507 | <0.0001 |
| NBG | Lesioned | DLS - GPe | L-DOPA / SKF82958 | -11.963078 | <0.0001 |
| NBG | Lesioned | DMS - GPe | SKF82958 / SUM | 1.03361577 | <0.0001 |
| NBG | Lesioned | DMS - GPe | SUM / L-DOPA | 3.73826085 | <0.0001 |
| NBG | Lesioned | DMS - GPe | L-DOPA / SKF82958 | -4.5897264 | <0.0001 |
| NBG | Lesioned | M1Tr - GPe | SKF82958 / SUM | 14.7512367 | <0.0001 |
| NBG | Lesioned | M1Tr - GPe | SUM / L-DOPA | -23.237089 | <0.0001 |
| NBG | Lesioned | M1Tr - GPe | L-DOPA / SKF82958 | 13.5249155 | <0.0001 |
| NBG | Lesioned | RFA - GPe | SKF82958 / SUM | 2.87330771 | <0.0001 |
| NBG | Lesioned | RFA - GPe | SUM / L-DOPA | -31.442745 | <0.0001 |
| NBG | Lesioned | RFA - GPe | L-DOPA / SKF82958 | 41.789436 | <0.0001 |

Abbreviations: Rostral forelimb area (RFA), primary motor cortex forelimb area (M1FL), primary motor cortex trunk area (M1Tr), dorsomedial striatum (DMS), dorsolateral striatum (DLS), and globus pallidus pars externa (GPe)

**Supplementary Table S7** Selected detailed statistical information from multiple comparison post hoc tests for phase synchrony. Find the complete statistical information in Supplemental statistical materials.

| **Phase synchrony** | | | | | | | | | |
| --- | --- | --- | --- | --- | --- | --- | --- | --- | --- |
| **Frequency range** | **Hemisphere** | **Structure**  **pair** | **Contrast** | **Estimate/ratio** | **SE** | **LCL** | **UCL** | **z-ratio** | **p-value** |
| NBG | Lesioned | DMS - GPe | LDOPA / SKF82958 | 0.09775 | 0.01 | 0.063 | 0.132 | 6.969 | <.0001 |
| NBG | Lesioned | DMS - GPe | LDOPA / SUM | 0.08704 | 0.01 | 0.051 | 0.123 | 5.901 | <.0001 |
| NBG | Lesioned | M1FL - DLS | LDOPA / SKF82958 | -0.09375 | 0.01 | -0.12 | -0.07 | -8.794 | <.0001 |
| NBG | Lesioned | M1FL - DLS | SKF82958 / SUM | 0.05917 | 0.01 | 0.033 | 0.086 | 5.443 | <.0001 |
| NBG | Lesioned | M1FL - DMS | LDOPA / SKF82958 | -0.09643 | 0.01 | -0.12 | -0.07 | -9.034 | <.0001 |
| NBG | Lesioned | M1FL - DMS | LDOPA / SUM | -0.04907 | 0.01 | -0.08 | -0.02 | -4.378 | 0.0001 |
| NBG | Lesioned | M1FL - DMS | SKF82958 / SUM | 0.04737 | 0.01 | 0.02 | 0.075 | 4.172 | 0.0002 |
| NBG | Lesioned | M1FL - M1Tr | LDOPA / SUM | -0.05664 | 0.01 | -0.08 | -0.03 | -5.05 | <.0001 |
| NBG | Lesioned | M1Tr - DMS | LDOPA / SKF82958 | -0.10562 | 0.02 | -0.15 | -0.07 | -6.425 | <.0001 |
| NBG | Lesioned | M1Tr - GPe | LDOPA / SUM | 0.08553 | 0.01 | 0.051 | 0.12 | 6.138 | <.0001 |
| NBG | Lesioned | M1Tr - GPe | SKF82958 / SUM | 0.06553 | 0.02 | 0.028 | 0.103 | 4.305 | 0.0001 |
| NBG | Lesioned | RFA - GPe | LDOPA / SKF82958 | 0.14252 | 0.02 | 0.102 | 0.183 | 8.694 | <.0001 |
| NBG | Lesioned | RFA - GPe | LDOPA / SUM | 0.13042 | 0.03 | 0.065 | 0.196 | 4.85 | <.0001 |
| NBG | Lesioned | RFA - M1FL | LDOPA / SKF82958 | 0.24104 | 0.01 | 0.222 | 0.26 | 30.559 | <.0001 |
| NBG | Lesioned | RFA - M1FL | LDOPA / SUM | 0.14283 | 0.01 | 0.121 | 0.165 | 15.994 | <.0001 |
| NBG | Lesioned | RFA - M1FL | SKF82958 / SUM | -0.09821 | 0.01 | -0.12 | -0.08 | -10.65 | <.0001 |
| NBG | Lesioned | RFA - M1Tr | LDOPA / SKF82958 | 0.10353 | 0.01 | 0.068 | 0.139 | 7.216 | <.0001 |
| NBG | Lesioned | DMS - GPe | LDOPA / SKF82958 | 0.09775 | 0.01 | 0.063 | 0.132 | 6.969 | <.0001 |
| NBG | Lesioned | DMS - GPe | LDOPA / SUM | 0.08704 | 0.01 | 0.051 | 0.123 | 5.901 | <.0001 |
| NBG | Lesioned | M1FL - DLS | LDOPA / SKF82958 | -0.09375 | 0.01 | -0.12 | -0.07 | -8.794 | <.0001 |

Abbreviations: Standard error (SE), Lower control limit (LCL), Upper control limit (UCL), Rostral forelimb area (RFA), primary motor cortex forelimb area (M1FL), primary motor cortex trunk area (M1Tr), dorsomedial striatum (DMS), dorsolateral striatum (DLS), and globus pallidus pars externa (GPe)

**References**

Andersson, M., Hilbertson, A., Cenci, M. A., 1999. Striatal fosB expression is causally linked with l-DOPA-induced abnormal involuntary movements and the associated upregulation of striatal prodynorphin mRNA in a rat model of Parkinson's disease. Neurobiol Dis 6, 461-474.
